# Non‐Relapse Mortality With Bispecific Antibodies in Hematologic Malignancies: A Dual Systematic Review and Pharmacovigilance Analysis

**DOI:** 10.1002/hon.70268

**Published:** 2026-10-16

**Authors:** Sohaib Irfan, Harshit Khosla, Saad Nasir, Manmeet Kaur, Tyler J. Varisco, Onyebuchi Ononogbu, Sharda Singh, Muhammad Bilal Abid

**Affiliations:** ^1^ The Aga Khan University School of Medicine Karachi Pakistan; ^2^ Department of Hematology/Oncology The University of Texas Houston McGovern School of Medicine Houston Texas USA; ^3^ Department of Lymphoma/Myeloma MD Anderson Cancer Center Houston Texas USA; ^4^ Center for Learning Health System Sciences University of Minnesota Minneapolis Minnesota USA; ^5^ University of Texas at Austin College of Pharmacy Austin Texas USA; ^6^ University of Houston College of Pharmacy Houston Texas USA; ^7^ Hematology and Medical Oncology Texas Tech University Health Sciences Center, School of Medicine Lubbock Texas USA

**Keywords:** bispecific antibodies, CRS, ICANS, infections, LBCL, multiple myeloma, neurotoxicity, non‐hodgkin lymphoma, non‐relapse mortality, safety

## Abstract

Bispecific antibodies (BsAbs) redirect T‐cells to tumor antigens in an MHC‐independent manner. These universally available, off‐the‐shelf products have demonstrated unprecedented efficacy in heavily pretreated patients with relapsed/refractory (R/R) hematological malignancies (HMs). The indefinite treatment duration associated with most BsAb products, coupled with neutropenia, B‐cell aplasia, and hypogammaglobulinemia result in significant immune‐related toxicities and infections. Hence, non‐relapse mortality (NRM) which represents the cumulative burden of treatment‐related toxicities, defined as death from any cause other than disease relapse or progression, is a patient‐centric endpoint across cellular therapy platforms. While recent NRM estimates with CAR T‐cell therapy have identified infections as major drivers of NRM, real‐world NRM estimates remain limited with BsAbs. To mitigate the limitations associated with each of the methods, we conducted a systematic review and meta‐analysis of the existing literature, supplemented concurrently by a pharmacovigilance analysis using the FAERS database, to estimate NRM with BsAbs and compare real‐world findings between commercially available BsAb products approved for relapsed/refractory multiple myeloma (RRMM) and NHL. The primary endpoint was NRM; secondary endpoints included CRS, ICANS, infections, and death. Teclistamab demonstrated a high toxicity burden across both analytic methods, with the highest ICANS incidences, significantly greater infection rates (particularly grade > 3), and a non‐negligible NRM estimate in the SRMA as well in FAERS. Glofitamab was associated with severe toxicities, including increased reporting odds for NRM and overall mortality. Mosunetuzumab showed lower pooled estimates and lower reporting odds, as seen in both analytic approaches. Low pooled incidences of infections, and ICANS were calculated in the SRMA. FAERS analyses also suggested reduced reporting odds for NRM and neurotoxicity. While trial‐level data and real‐world reporting largely converged for severe toxicities, divergences in other agents underscore the importance of continuous post‐marketing surveillance. Given the risks associated with prolonged treatment, future protocols must prioritize infection management and adopt NRM as a standardized endpoint to guide appropriate therapeutic selection and sequencing.

## 1 Introduction

Bispecific antibodies (BsAbs) connect CD3 on endogenous T‐cells to tumor‐associated antigens (TAA) on cancer cells, without the need for ex vivo cell manipulation, and have emerged as an accessible and efficacious therapy for patients with hematologic malignancies [1, 2].

Traditionally, BsAbs have been used primarily for relapsed or refractory multiple myeloma (RRMM) (targeting BCMA and GPRC5D) and R/R B‐cell non‐Hodgkin lymphoma (NHL) (targeting CD20). BsAbs are currently undergoing active investigation in earlier lines of treatment for cancers as well as for non‐malignant conditions, such as autoimmune and neurologic diseases [3, 4].

BsAbs work by activating T‐cell's cytotoxicity mechanisms. Immune toxicities such as cytokine release syndrome (CRS) and immune effector cell–associated neurotoxicity syndrome (ICANS) occur less frequently and with reduced severity compared with chimeric antigenic T cell therapy (CAR‐T) [5].

The indefinite treatment duration associated with most BsAbs products, coupled with neutropenia, B‐cell aplasia, and hypogammaglobulinemia results in significant immune‐related toxicities and infections [6, 7, 8]. Hence, non‐relapse mortality (NRM), defined as death from any cause other than disease relapse or progression, is a patient‐centric endpoint across cellular therapy platforms. However, with bispecific antibodies (BsAbs), accurately capturing NRM remains elusive because it reflects the cumulative burden of treatment‐related toxicities [9].

To evaluate the incidence and burden of these outcomes, we conducted a systematic review and meta‐analysis of the existing literature, supplemented concurrently by a pharmacovigilance analysis, to estimate NRM with BsAbs and compare real‐world findings between commercially available BsAbs products.

## 2 Methods

### 2.1 Systematic Review and Meta‐Analysis

#### 2.1.1 Search Strategy and Study Selection

Following the PRISMA 2020 guidelines, we conducted a systematic review and meta‐analysis. A comprehensive literature search was performed in PubMed, Embase, and major clinical trial registries up to September 30, 2024. The search combined controlled vocabulary and free‐text terms related to bispecific antibodies (e.g., ‘teclistamab,’ ‘elranatamab,’ ‘talquetamab,’ ‘glofitamab,’ ‘epcoritamab,’ ‘mosunetuzumab’), disease subtypes (e.g., ‘diffuse large B‐cell lymphoma (DLBCL),’ ‘follicular lymphoma (FL),’ ‘mantle cell lymphoma (MCL),’ ‘multiple myeloma (MM)’), and outcomes (‘non‐relapse mortality,’ ‘causes of death,’ ‘adverse events,’ ‘toxicity,’ ‘survival’). No filters were applied for the study.

Screening of titles, abstracts, and full texts and selection process given in the PRISMA flow diagram (Figure 1).

**FIGURE 1:**
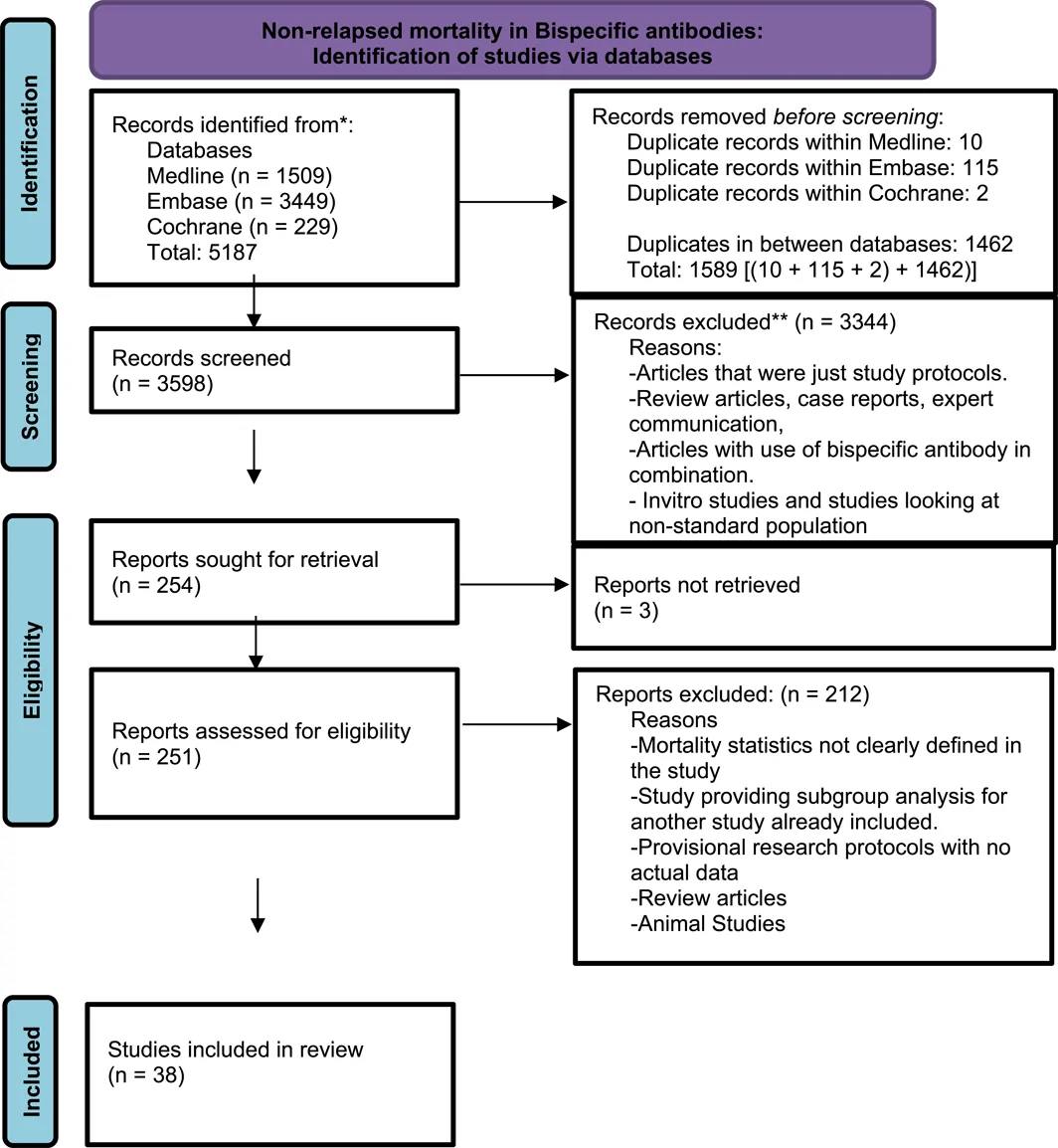
Prisma diagram.

#### 2.1.2 Eligibility Criteria

The inclusion criteria included observational studies and interventional trials of adult patients (≥ 18 years) who were diagnosed with R/R LBCL, MCL, FL, MM, treated with an FDA‐approved bispecific antibody, and reported NRM. Case reports, reviews, and animal studies were excluded.

#### 2.1.3 Data Extraction and Quality Assessment

A pre‐specified template was used for the data extraction process (Table 1). The template included sample size, author names, disease subtypes, BsAbs type, follow‐up duration, and prior lines of therapy (LOT). The outcomes included the following.

- NRM (defined as death without preceding relapse or progression)
- Infection rates
- Fatal infection rates
- Grade ≥ 3 infection rates (graded per CTCAE v5.0)
- Cytokine release syndrome (CRS)
- Grade ≥ 3 CRS (graded per the ASTCT criteria)
- Immune effector cell‐associated neurotoxicity syndrome (ICANS) (graded per the ASTCT criteria)

**TABLE 1:** Characteristics of studies included.

Authors	Year	Underlying dx	BsAb products	Cohort size (*n*)	Number of PLOT:	Follow up in month	NRM *n* (%)	Infections *n* (%)	Infections (*G* ≥ 3) *n* (%)	Fatal infections *n* (%)	CRS, *n* (%)	CRS (*G* ≥ 3), *n* (%)	iCANS, *n* (%)
Khouderchah et al. [10]	2024	MM	Tec	4	NA	NA	2 (50)	2 (50)	0 (0)	0 (0)	1 (25)	0 (0)	1 (25)
Gong et al. [11]	2023	MM	Tec	1314	NA	NA	92 (7)	36 (3)	36 (3)	36 (3)	NA	0 (0)	0 (0)
Rodriguez‐Otero et al. [12]	2023	MM	Talq	339	NA	15.9	3 (1)	NA	NA	2 (1)	NA	NA	NA
Janardan et al. [13]	2023	MM	NA	15	NA	NA	NA	NA	9 (60)	7 (47)	NA	NA	NA
Jourdes et al. [14]	2024	MM	Tec,talq	229	4	NA	5 (2)	160 (70)	123 (54)	20 (9)	138 (60)	37 (16)	12 (5)
Nath et al. [15]	2023	MM	NA	55	6	4.3	39 (70)	55 (100)	21 (38)	4 (7)	NA	NA	NA
Uttervall et al. [16]	2024	MM	Tec, talq	58	7	15.1	2 (4)	57 (98)	NA	4 (7)	NA	NA	NA
Raje et al. [17]	2023	MM	El	123	NA	6.4	9 (7.6)	58 (47)	32 (26)	7 (6)	48 (39)	NA	5 (4)
Lesokhin et al. [18]	2023	MM	El	187	5	14.7	7 (4)	86 (46)	49 (26)	8 (4)	67 (36)	0 (0)	4 (2)
Venkatesh et al. [19]	2023	MM	Tec	22	6	3.1	0 (0)	NA	NA	0 (0)	9 (41)	NA	5 (23)
Moreau et al. [20]	2022	MM	Tec	165	5	14.1	27 (16.4)	126 (76)	74 (45)	19 (12)	119 (72)	1 (1)	24 (15)
Chari et al. [21]	2022	MM	Talq	232	6	11.7	2 (1)	29 (12)	5 (2)	2 (1)	108 (47)	6 (3)	8 (3)
Podder et al. [22]	2024	MM	El	6	5	NA	4 (60)	5 (83)	5 (83)	3 (50)	5 (83)	0 (0)	0 (0)
Donk et al. [23]	2021	MM	Talq	28	5.5	6.2	NA	9 (32)	1 (4)	NA	22 (79)	4 (14)	2 (7)
Lesokhin et al. [24]	2022	MM	El	60	5	NA	2 (3.3)	28 (47)	11 (18)	1 (2)	35 (58)	0 (0)	2 (3)
Dima et al. [25]	2023	MM	Tec	102	> 3	3.2	0 (0)	29 (28)	14 (14)	0 (0)	66 (65)	1 (1)	15 (15)
Krisnan et al. [26]	2021	MM	Talq	28	5.5	6.2	NA	9 (32)	1 (4)	0 (0)	22 (79)	1 (4)	2 (7)
Gordon et al. [27]	2023	MM	Tec	45	NA	NA	NA	4 (9)	0 (0)	0 (0)	25 (55)	2 (4)	13 (29)
Uttervall et al. [28]	2022	MM	Tec	17	9	NA	0 (0)	6 (35)	6 (35)	0 (0)	9 (53)	1 (6)	1 (6)
Donk et al. [29]	2023	MM	Tec	165	> 2	23	8 (4.8)	132 (80)	91 (55)	1 (1)	119 (72)	1 (1)	5 (3)
Mohan et al. [30]	2024	MM	Tec	110	> 2	3.5	2 (1.82)	44 (40)	27 (25)	1 (1)	62 (56)	4 (4)	12 (11)
Stepanovic et al. [31]	2023	MM	Tec	33	> 2	4.1	4 (12)	16 (48)	NA	3 (9)	12 (36)	0 (0)	5 (15)
Dong et al. [32]	2024	NHL	Ep	26	4	4	1 (4)	1 (4)	1 (4)	1 (4)	8 (31)	0 (0)	0 (0)
Giatra et al. [33]	2022	NHL	Glof	5	NA	NA	2 (40)	2 (40)	2 (40)	2 (40)	NA	NA	NA
Hutchings et al. [34]	2021	NHL	Ep	68	NA	NA	0 (0)	NA	NA	NA	40 (59)	0 (0)	NA
Budde et al. [35]	2024	NHL	Mon	229	3	42	NA	NA	NA	NA	NA	NA	NA
Gurion et al. [36]	2023	NHL	Glof	35	5	34	6 (17)	NA	6 (17)	6 (17)	13 (37)	0 (0)	2 (6)
Thieblemont et al. [37]	2022	NHL	Ep	157	3	10.7	3 (2)	71 (45)	23 (15)	1 (1)	78 (50)	4 (3)	10 (6)
Budde et al. [38]	2021	NHL	Mon	129	≥ 2	11.9	8 (6.2)	20 (16)	0 (0)	1 (1)	35 (27)	0 (0)	13 (10)
Dickinson et al. [39]	2022	NHL	Glof	155	3	12.6	8 (5)	59 (38)	23 (15)	7 (5)	97 (63)	5 (3)	12 (8)
Karimi et al. [40]	2023	NHL	Ep	157	3	20	NA	NA	NA	NA	80 (51)	82 (52)	9 (6)
Puertas et al. [41]	2022	NHL	Mon	22	3	14.3	NA	12 (55)	NA	NA	15 (68)	NA	NA
Olszewski et al. [42]	2022	NHL	Mon	54	NA	23.3	2 (3)	2 (4)	2 (4)	2 (4)	14 (26)	0 (0)	0 (0)
Cheah et al. [43]	2023	NHL	Mon	20	2.5	NA	0 (0)	7 (35)	0 (0)	0 (0)	13 (65)	1 (5)	2 (10)
Lewis et al. [44]	2023	NHL	Ep	157	> 3	10.7	NA	NA	NA	NA	78 (50)	4 (3)	10 (6)
Budde et al. [45]	2023	NHL	Mon	47	> 1	2.5	1 (2)	12 (26)	3 (6)	1 (2)	11 (23)	0 (0)	0 (0)
Falchi et al. [46]	2023	NHL	Mon	43	1	6	1 (2.3)	10 (23)	2 (5)	1 (2)	20 (47)	2 (5)	0 (0)
Clausen et al. [47]	2021	NHL	Ep	46	> 1	10.2	0 (0)	NA	NA	0	27 (59)	0 (0)	4 (9)

Abbreviations: BsAb, Bispecific Antibodies; CRS, Cytokine release syndrome; Dx, Diagnosis; El, Elranatamab; EP, Epcoritamab; G ≥ 3, Grade 3 and above; Glof, Glofitamab; ICANS, Immune Effector Cell–Associated Neurotoxicity Syndrome; MM, Multiple Myeloma; Mon, Mosunetuzumab; NA, Not Available; NHL, Non‐Hodgkin Lymphoma; NRM, Non‐Relapse Mortality; PLOT, previous lines of therapy; Talq, Talquetamab; Tec, Teclistamab.

Two reviewers independently assessed the quality of each included study. Because the included evidence base consisted almost entirely of single‐arm trials and single‐cohort real‐world series rather than comparative studies, with a substantial proportion reported as conference abstracts, study quality was used to inform the certainty attached to the pooled and agent‐level estimates rather than as a basis for study exclusion.

#### 2.1.4 Statistical Analysis

Data were analyzed in R (v.4.3.1), using the metafor and meta packages. NRM point estimates were determined by performing random‐effect meta‐analyses of single proportions utilizing a generalized linear mixed model. This method was chosen to account for between‐study heterogeneity. Proportions were reported with 95% confidence intervals (CIs). Because non‐relapse mortality is a time‐dependent outcome, pooled proportions reflect cumulative event burden over variable follow‐up durations rather than time‐adjusted risk estimates. Studies reporting zero events were explicitly included in the analysis to prevent upward bias in the pooled incidence rates, ensuring the estimates accurately reflect the true cumulative burden. Subgroup analyses were performed by individual BsAbs products with/without combination cohorts and disease subtypes. Heterogeneity was assessed with the I^2^ statistic. Values greater than 50% for the I^2^ statistic were considered substantial. Publication bias was assessed using funnel plots and Egger's test, where more than 10 studies were available.

To assess whether cumulative NRM was influenced by observation time, we performed a random‐effects meta‐regression of NRM proportion on median follow‐up duration among studies reporting it. We stratified NRM by regimen type (continuous‐until‐progression vs. fixed‐duration), classified per approved product labeling. Studies not reporting follow‐up were retained in the primary pooled estimate but excluded from the meta‐regression (available‐case analysis). A sensitivity analysis restricted the pooled NRM to follow‐up–reporting studies. Studies for which the specific BsAbs was unspecified contributed to overall and disease‐level estimates but were excluded from agent‐level analyses. Analyses were performed using R software version 4.3.2.

### 2.2 FAERS Database Analysis

We queried the FDA Adverse Event Reporting System (FAERS) post‐marketing surveillance database for adverse event (AE) reports across 6 commercially available BsAbs products (Supporting Information S1: eMethods in the appendix). In accordance with the Common Rule, this study was exempt from ethics review and informed consent, as publicly accessible anonymized data were used. We followed STROBE reporting guidelines.

The primary endpoint was NRM; secondary endpoints included CRS, ICANS, infections, and death coded at the preferred term level in the Medical Dictionary for Regulatory Activities (MedDRA) classification. Because FAERS does not capture adjudicated relapse or progression status, non‐relapse mortality could not be directly ascertained; therefore, NRM in the FAERS analysis represents toxicity‐associated mortality inferred by exclusion of reports coded as disease progression.

Descriptive statistics included frequencies to present various demographic factors and the occurrence of AEs across different antibodies. Disproportionality analysis was performed to detect reporting of specific drug‐AE combinations in comparison to the reference group. Odds ratios (ORs) with 95% CIs were calculated, and separate OR estimates were reported for each BsAb. We further compared the odds of experiencing a BsAb's specific AE to the odds of experiencing the same AE with any of the other BsAbs in the dataset. Pearson correlation coefficients were calculated to measure associations between different AEs, irrespective of the specific drug administered. Analyses were performed using R and SAS statistical software. Head to head comparisoTn in addition to diverging and converging signals for both approaches are summarized in (Table 2, 3, 4).

**TABLE 2:** A side‐by‐side comparison of the incidence rates from the meta‐analysis and the reporting odds ratios from the FAERS database.

Bispecific antibodies	NRM		Infection		CRS		ICANS	
	SRMA	FAERS	SRMA	FAERS	SRMA	FAERS	SRMA	FAERS
Elranatamab	9% (1–54)	0.96 (0.75–1.23)	47% (39–55)	1.48 (1.22–1.80)	46% (26–67)	0.62 (0.49–0.79	3% (1–7)	1.18 (0.78–1.80)
Teclistamab	4% (1–14)	1.55 (1.29–1.85)	35% (14–64)	0.95 (0.80–1.12)	58% (47–68)	0.69 (0.58–0.83)	7% (2–24)	2.69 (2.03–3.58)
Talquetamab	NA	0.33 (0.23–0.46)	22% (5–58)	0.44 (0.35–0.55)	67% (23–93)	0.43 (0.34–0.55)	4% (1–13)	1.05 (0.70–1.59)
Glofitamab	12% (1–62)	1.24 (1.03–1.49)	NA	0.77 (0.65–0.90)	NA	1.63 (1.40–1.91)	NA	0.75 (0.51–1.09)
Epcoritamab	1% (0–4)	1.17 (1.01–1.35)	NA	1.40 (1.24–1.58)	51% (46–56)	1.48 (1.30–1.68)	6% (4–10)	0.74 (0.56–0.98)
Mosunetuzumab	4% (2–9)	0.47 (0.34–0.65)	22% (9–44)	0.96 (0.77–1.18)	40% (23–60)	0.79 (0.63–1.00)	1% (0–42)	0.08 (0.02–0.34)

Abbreviations: CRS, Cytokine Release syndrome; FAERS, FDA Adverse Event Reporting System; ICANS, Immune Effector Cell‐Associated neurotoxicity Syndrome; NA, Not available (Less number of studies for meta‐analysis); NRM, Non‐Relapse Mortality; SRMA, Systematic Review and Meta analysis.

**TABLE 3:** Comparison of clinical outcomes between systematic review/meta‐analysis and FAERS pharmacovigilance.

Outcome	Systematic review & meta‐analysis (incidence)	FAERS pharmacovigilance (reporting association)
Non‐relapse mortality (NRM)	Overall: 4% (95% CI, 2%–7%) Range: 1% (epcoritamab)–12% (glofitamab)	Highest reporting odds: Teclistamab (ROR 1.55), glofitamab (ROR 1.24) Lowest reporting odds: Talquetamab (ROR 0.33), mosunetuzumab (ROR 0.47)
Infections (any grade)	Overall: 40% (95% CI, 26%–56%) Highest: Elranatamab (47%)	Highest reporting odds: Elranatamab (ROR 1.48), epcoritamab (ROR 1.40) Lowest reporting odds: Talquetamab (ROR 0.44
Cytokine release syndrome (CRS)	Overall: 52% (95% CI, 46%–58%) Highest: Talquetamab (67%)	Highest reporting odds: Glofitamab (ROR 1.63), epcoritamab (ROR 1.48) Lowest reporting odds: Talquetamab (ROR 0.43)
ICANS (neurotoxicity)	Overall: 5% (95% CI, 3%–8%) Highest: Teclistamab (7%)	Highest reporting odds: Teclistamab (ROR 2.69) Lowest reporting odds: Mosunetuzumab (ROR 0.08)
Mortality specifics	Fatal infection: 2%–3% Grade ≥ 3 infection: 12%	Highest mortality within AE Reports Infection: Glofitamab (39.2%) CRS: Glofitamab (26.1%) ICANS: Mosunetuzumab (50%)

**TABLE 4:** Key discrepancies and converging signals between clinical trials (SRMA) and post‐marketing surveillance (FAERS).

Outcome	Clinical trial signal (SRMA)	Real‐world signal (FAERS)
Non‐relapse mortality (NRM)	Highest risk: Glofitamab (12%)	Highest reporting odds: Teclistamab (ROR 1.55)
CRS incidence	Highest: Talquetamab (67%)	Lowest/Protective reporting odds: Talquetamab (ROR 0.43)
Infection risk	High: Elranatamab (47%) and teclistamab (35%)	High reporting odds: Elranatamab (ROR 1.48) and epcoritamab (ROR 1.40)
Neurotoxicity (ICANS)	Highest: Teclistamab (7%)	Highest reporting odds: Teclistamab (ROR 2.69)
Fatal outcomes	Highest fatal infections: Glofitamab (12%)	Highest mortality from AEs: Glofitamab

## 3 Results

### 3.1 Study Characteristics

#### 3.1.1 Meta‐Analysis

In this systematic review and meta‐analysis, 38 studies met the inclusion criteria, including 22 MM studies and 16 NHL studies, with a total of 4687 patients (Table 1). Thirteen studies reported CD20 as the target antigen, while 20 studies reported BCMA, and 5 studies evaluated both. Agent‐level data were reported for teclistamab (10 studies), epcoritamab (6), mosunetuzumab (7), elranatamab (4), talquetamab (4), and glofitamab (3). The median follow‐up duration was 12.3 months (range, 2.5–42 months) with a median prior lines of therapy (LOT) of 3 (range, 1–9).

#### 3.1.2 FAERS Database

Between September 1, 2022 (Q3) and September 30, 2024 (Q3), the duration during which all currently available BsAbs were approved by the FDA for NHL and MM, we identified 15,820,932 total AE reports in FAERS. Among these, 5066 reports were for the drugs of interest, reporting a total of 4157 AEs across 3125 reports. The 5066 unique patients included 1779 females (35.1%) and 2273 males (44.9%) with a median age of 68 years (IQR: 60–74) (Supporting Information S1: Table S1). There were 1028 reports of death (20.3%), 139 of which were NRM (2.7%). There were 1365 reports of CRS (26.9%), 237 of ICANS (4.7%), and 1525 of infections (30.1%) (Supporting Information S1: Table S2). The outcomes of each AE, stratified by the BsAbs product, their co‐occurrence, and correlation matrices, are given in Supporting Information S1: Tables S2–S3 and Supporting Information S1: Figures S1–S2, respectively.

When stratifying AE reports by MM BsAbs products, 595 AEs were reported with teclistamab, of which 166 were CRS (27.9%), 75 were ICANS (12.6%), and 228 were infections (38.3%) (Table 1). With elranatamab, 374 AEs were reported, of which 93 were CRS (24.8%), 26 were ICANS (6.9%), and 183 were infections (48.9%). Additionally, 219 AEs were reported with talquetamab, of which 81 were CRS (36.9%), 27 were ICANS (12.3%), and 93 were infections (42.4%) (Table 1).

Similarly, for NHL BsAbs products, 741 AEs were reported with glofitamab, of which 307 were CRS (41.4%), 32 were ICANS (4.3%), and 222 were infections (29.9%). With epcoritamab, 1671 AEs were reported, of which 615 were CRS (36.8%), 75 were ICANS (4.4%), and 668 were infections (39.9%). For mosunetuzumab, 271 AEs were reported, of which 103 were CRS (38%), 2 were ICANS (0.7%), and 131 were infections (48.3%). Outcomes of AEs stratified by BsAbs products are given in Supporting Information S1: Figures S3–S4.

#### 3.1.3 Non‐Relapse Mortality (NRM)

##### 3.1.3.1 Systematic Review and Meta‐Analysis

With the meta‐analysis, the overall NRM point estimate across all studies (including combination cohorts) was 4% (95% CI, 2%–7%) (Figure 2). With individual BsAb products (no combination cohorts), the NRM point estimate was reported at 4% (95% CI, 2%–6%) (Figure 3). When stratified by disease, NHL had a lower NRM (3%) compared to MM (4%–5%) (Figure 2and 3). At the BsAb agent level, glofitamab and elranatamab displayed a notably elevated NRM point estimate of 12% and 9% respestively; however, this finding must be interpreted with caution due to the substantial uncertainty of the estimate. Epcoritamab showed the lowest NRM point estimate at 1% (95% CI, 0%–4%). Mosunetuzumab and teclistamab both reported NRM point estimates at 4% (Figure 4).

**FIGURE 2:**
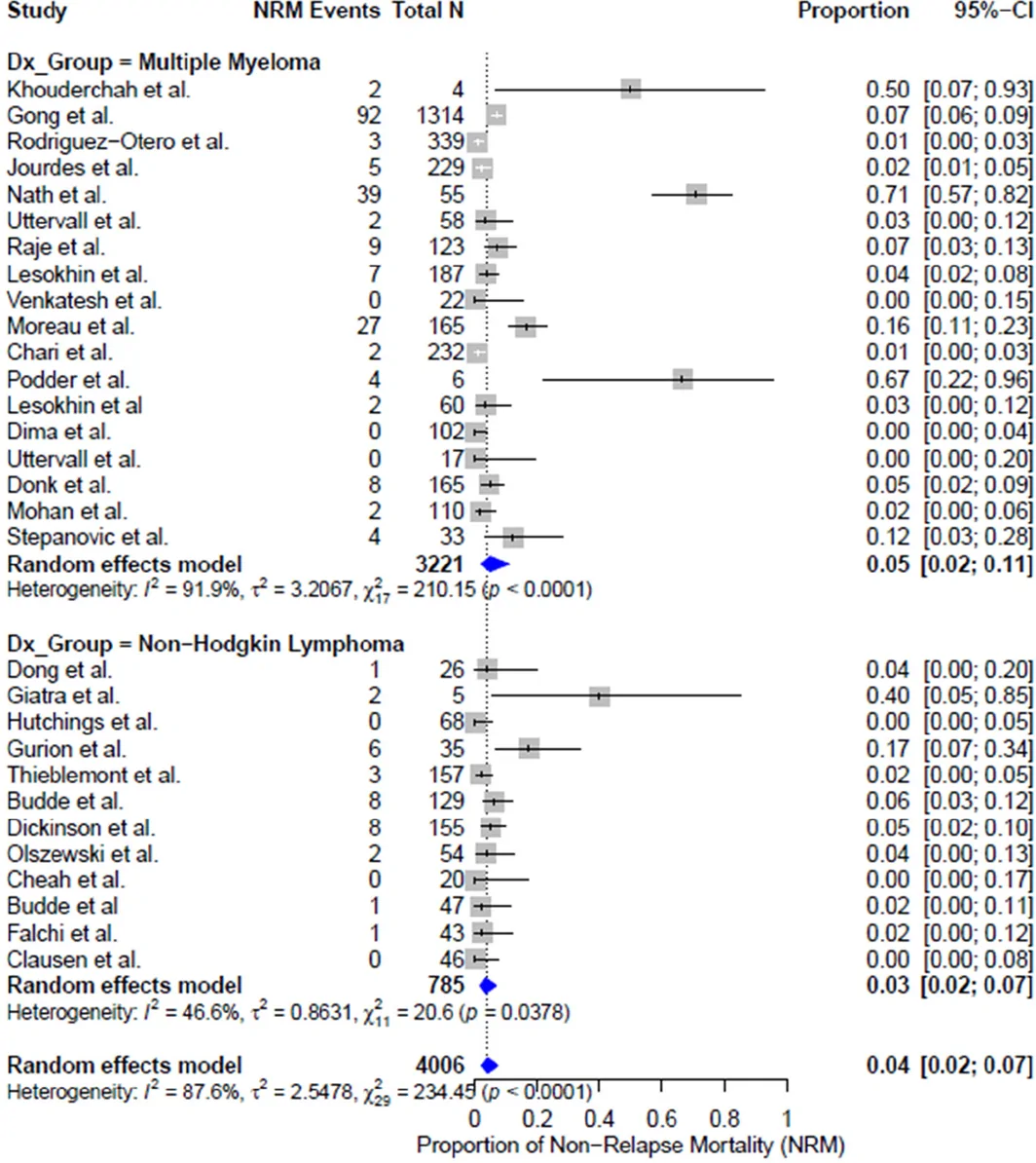
Forest plot showing pooled estimates of non‐relapse mortality (NRM) across all included studies and cohorts.

**FIGURE 3:**
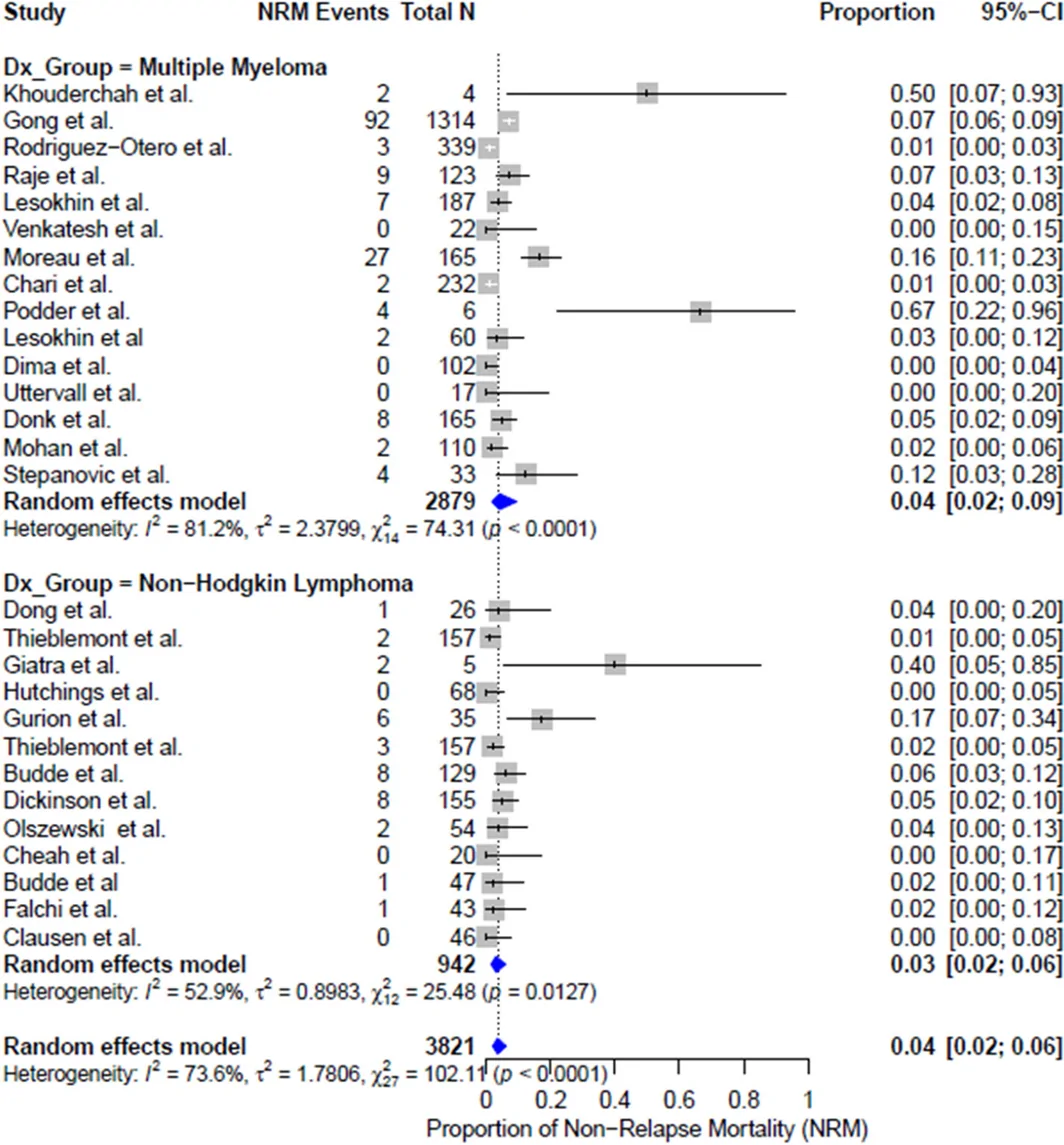
Forest plot of non‐relapse mortality (NRM) proportions across included studies excluding combination cohort studies, stratified by disease group (Multiple Myeloma and Non‐Hodgkin Lymphoma).

**FIGURE 4:**
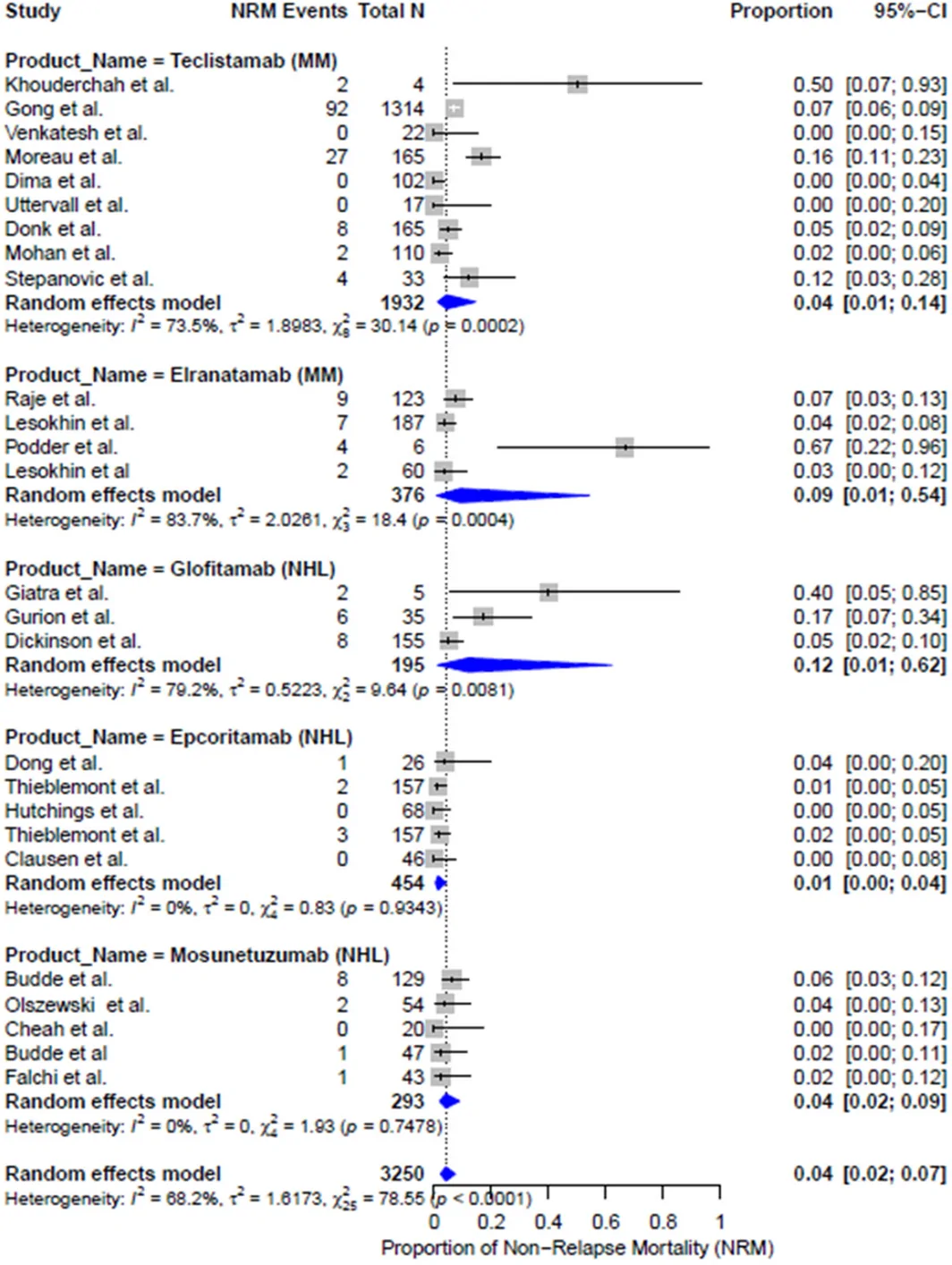
Forest plot depicting non‐relapse mortality (NRM) proportions stratified by bispecific antibody product.

#### 3.1.4 FAERS Pharmacovigilance

FAERS database analysis showed that of the 6 BsAb products, teclistamab was associated with the highest reporting odds for NRM (ROR = 1.55 [95% CI, 1.29–1.86]), followed by glofitamab (ROR = 1.24 [95% CI, 1.03–1.49]) and epcoritamab (ROR = 1.17 [95% CI, 1.01–1.35]). Other BsAbs were less likely to be associated with NRM, including mosunetuzumab (ROR = 0.47 [95% CI, 0.34–0.65]) and talquetamab (ROR = 0.33 [95% CI, 0.23–0.46]) (Figure 23). In congruence with NRM, glofitamab was also associated with greater odds for overall mortality (ROR = 1.61 [95% CI, 1.36–1.91]), as was teclistamab (ROR = 1.30 [95% CI, 1.08–1.55]), while talquetamab (ROR = 0.28 [95% CI, 0.20–0.39]) and mosunetuzumab (ROR = 0.76 [95% CI, 0.59–0.98]) were associated with lower reporting odds for overall mortality (Figure 23).

Across the 21 studies reporting follow‐up, meta‐regression showed no significant association between follow‐up duration and NRM (odds ratio 1.03 per additional month; 95% CI 0.95–1.12; *p* = 0.49). This argues against observation time as a primary driver of between‐agent differences. Pooled NRM did not differ significantly between continuous and fixed‐duration regimens (3.1% vs. 5.6%; *p* = 0.43). The numerically higher fixed‐duration estimate, driven by glofitamab, is opposite to the direction expected if longer cumulative exposure were the principal driver. Restricting the pooled estimate to follow‐up–reporting studies left it materially unchanged (3.6% vs. 4.0%). Agent‐level NRM with corresponding follow‐up availability is presented in Supporting Information S1: Table S4.

In a sensitivity analysis excluding very small studies, the overall pooled NRM estimate was essentially unchanged: 3.1% (95% CI, 1.7%–5.6%) excluding studies with fewer than 10 patients and 3.3% (95% CI, 1.8%–5.9%) excluding those with fewer than 20 patients, compared with 4.0% (95% CI, 2.1%–7.4%) in the primary analysis. The wide agent‐level confidence intervals were attributable to individual small cohorts; excluding a 6‐patient elranatamab cohort narrowed its estimate from 9.1% (95% CI, 1%–54%) to 4.9% (95% CI, 3.1%–7.6%), and excluding a 5‐patient glofitamab cohort narrowed its estimate from 12.0% (95% CI, 4.2%–29.7%) to 8.6% (95% CI, 3.6%–19.4%).

### 3.2 Infection Rates

#### 3.2.1 Systematic Review and Meta‐Analysis

The pooled infection rate across all studies (including combination cohorts) was 40% (95% CI, 26%–56%) (Figure 5). When restricting to only individual products (no combination cohorts), the incidence was reduced to 32% (95% CI, 22%–43%) (Figure 6). Stratified by disease, high infection rates (∼37%–51%) were noted for MM patients compared with NHL patients (24%). The highest infection rates were observed for elranatamab at 47% (95% CI, 39%–55%) and teclistamab at 35% (95% CI, 14%–64%). Conversely, mosunetuzumab and talquetamab reported the lowest rates at 22% (95% CI, 9%–44%) and 22% (95% CI, 5%–58%), respectively (Figure 7).

**FIGURE 5:**
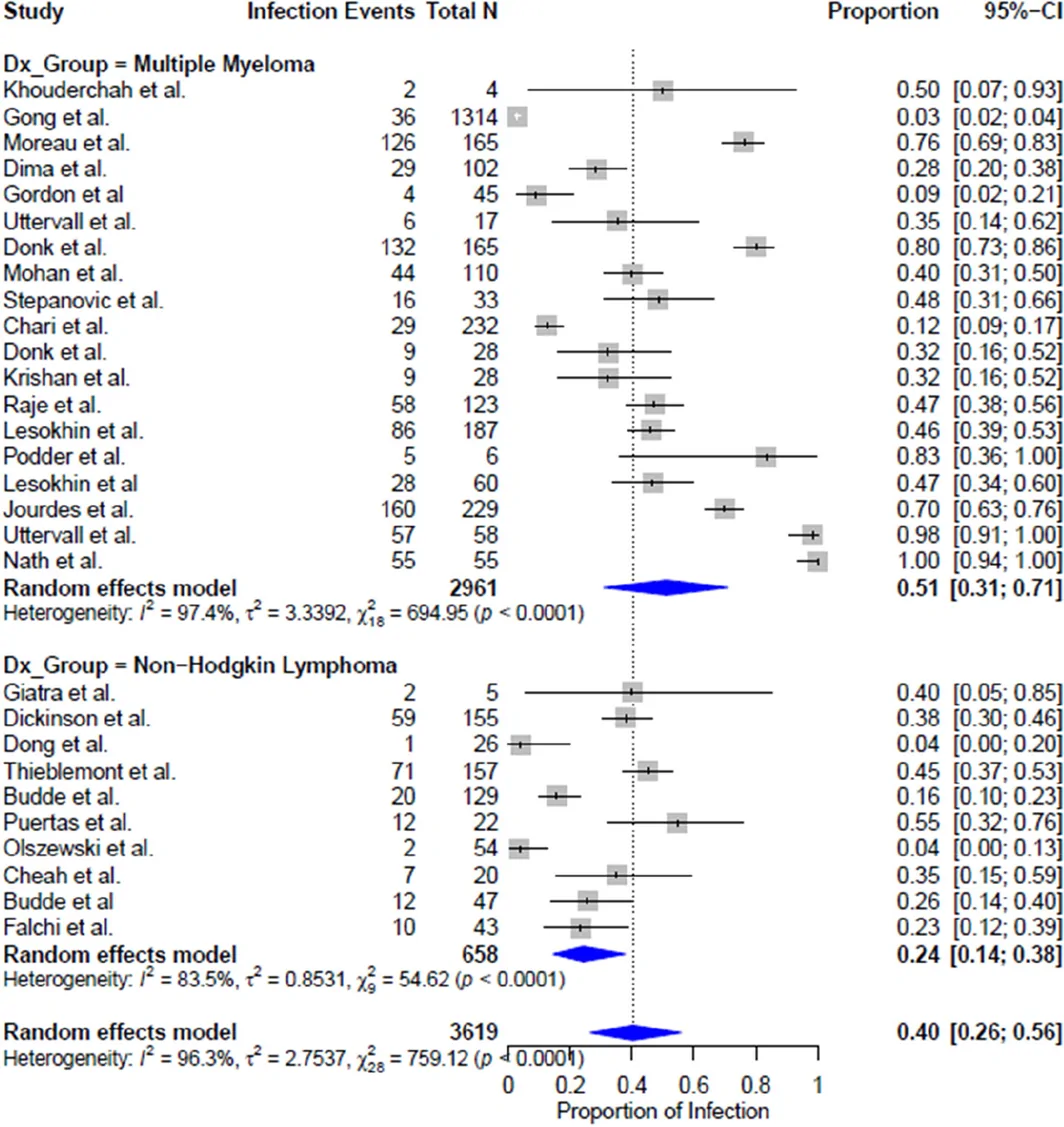
Odds ratio for adverse events by drugs.

**FIGURE 6:**
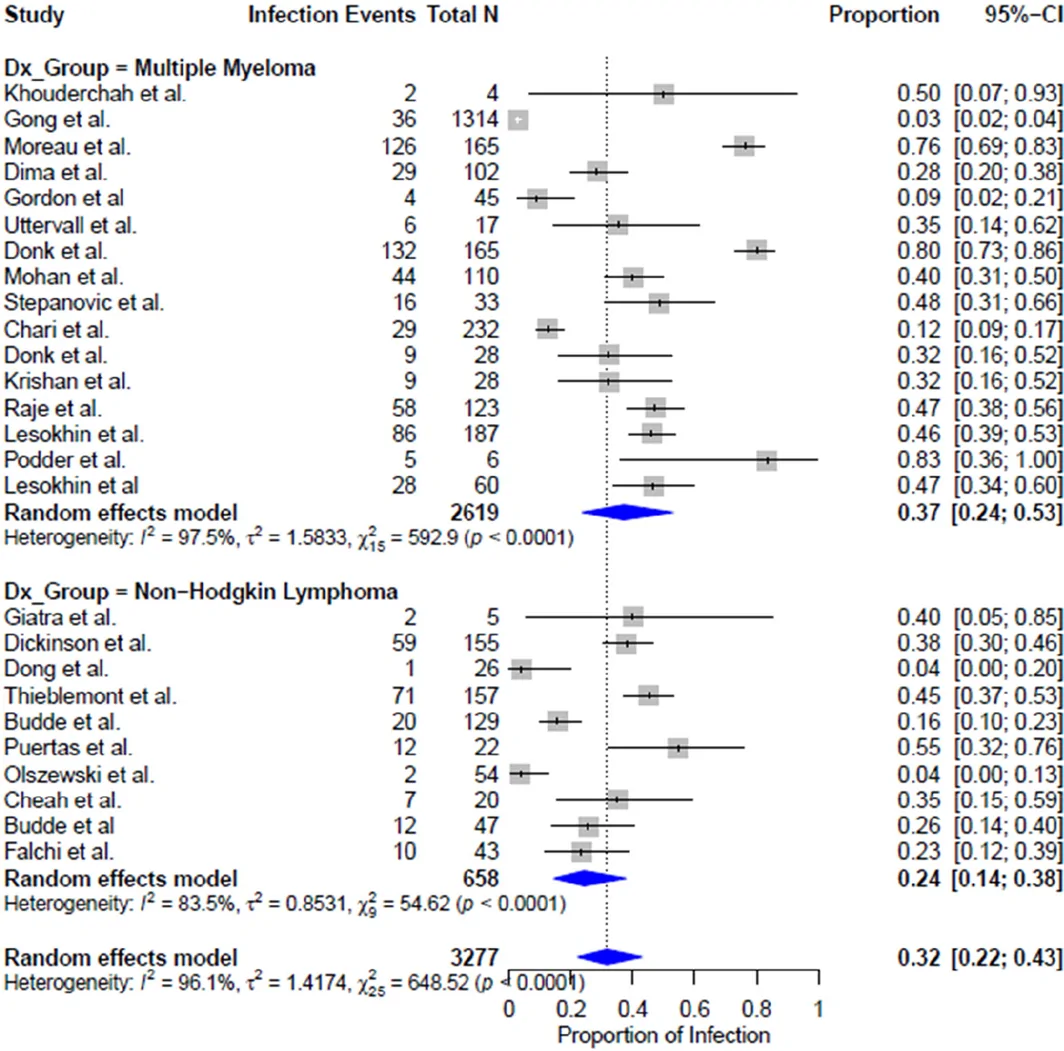
Forest plot of infection rates across included studies excluding combination cohort studies, stratified by disease group (Multiple Myeloma and Non‐Hodgkin Lymphoma).

**FIGURE 7:**
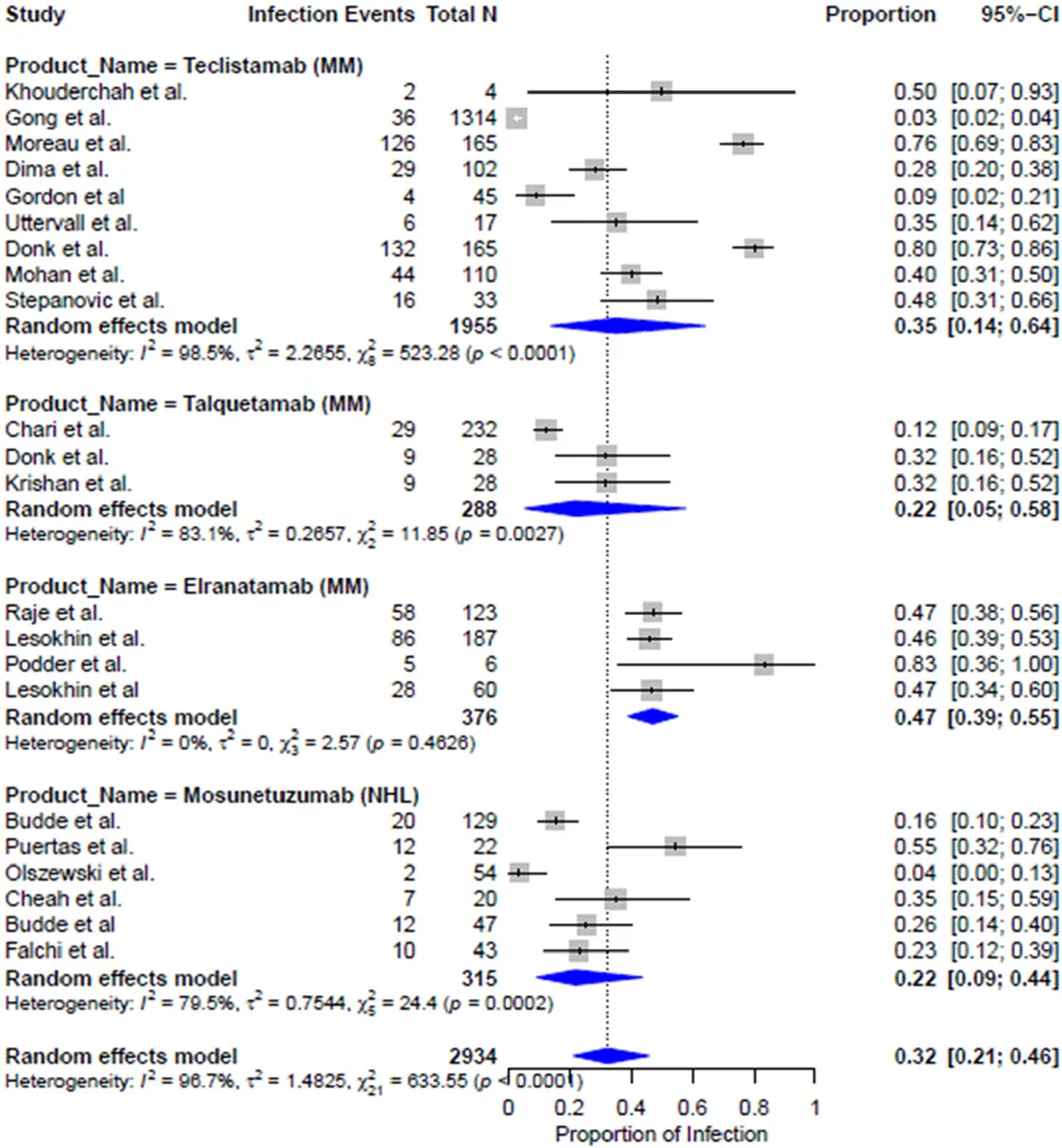
Forest plot depicting infection rates stratified by bispecific antibody product.

#### 3.2.2 FAERS Pharmacovigilance

Increased reporting odds for infections were observed with elranatamab (ROR = 1.48 [95% CI, 1.22–1.80]) and epcoritamab (ROR = 1.40 [95% CI, 1.24–1.58]), while talquetamab and glofitamab were associated with reduced odds (ROR = 0.44 [95% CI, 0.35–0.55] and ROR = 0.77 [95% CI, 0.65–0.90], respectively) (Figure 23).

### 3.3 Fatal Infections

#### 3.3.1 Systematic Review and Meta‐Analysis

Overall, fatal infections were uncommon. Pooled estimates of 2%–3% were observed across the analyses. The overall fatal infection point estimate, when subgrouped by disease, was 3% for NHL and 2%–3% for MM (Figure 8, Figure 9). Agent‐level analysis revealed fatal infection estimate of 12% (95% CI, 1%–65%) with glofitamab, 7% with elranatamab, 1% for teclistamab, and 2% for mosunetuzumab (Figure 10).

**FIGURE 8:**
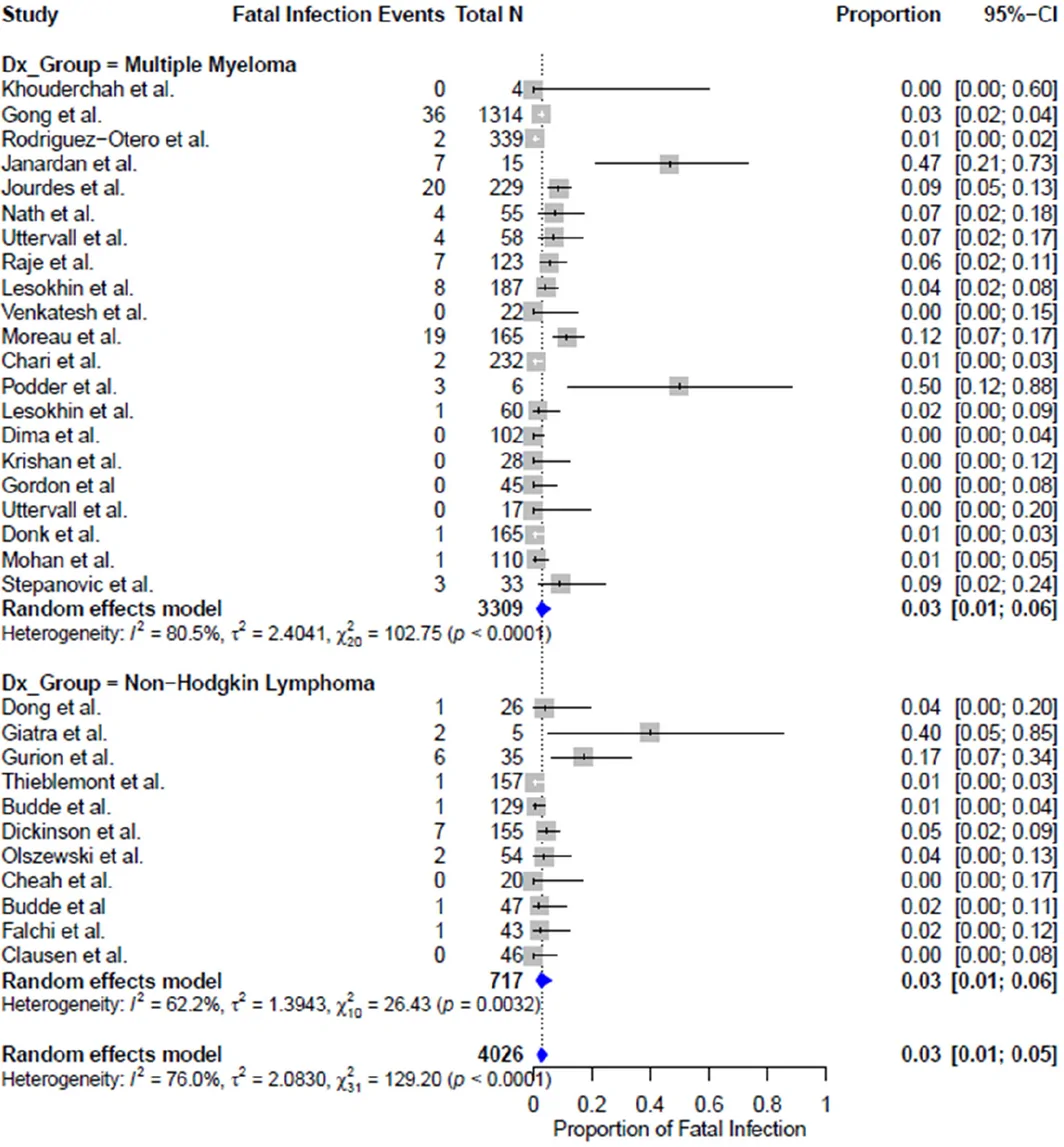
Forest plot showing pooled estimates of infection rates across all included studies and cohorts.

**FIGURE 9:**
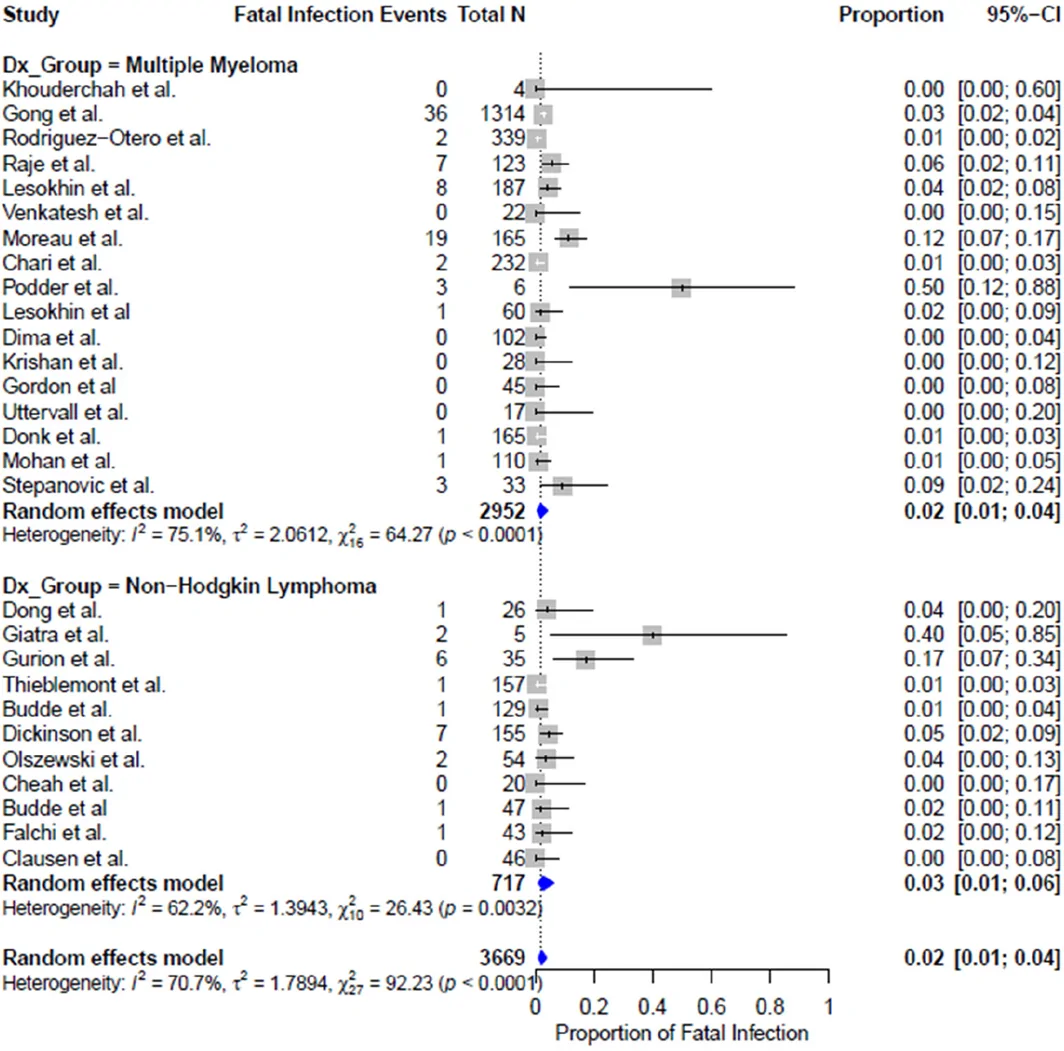
Forest plot of fatal infection rates across included studies excluding combination cohort studies, stratified by disease group (Multiple Myeloma and Non‐Hodgkin Lymphoma).

**FIGURE 10:**
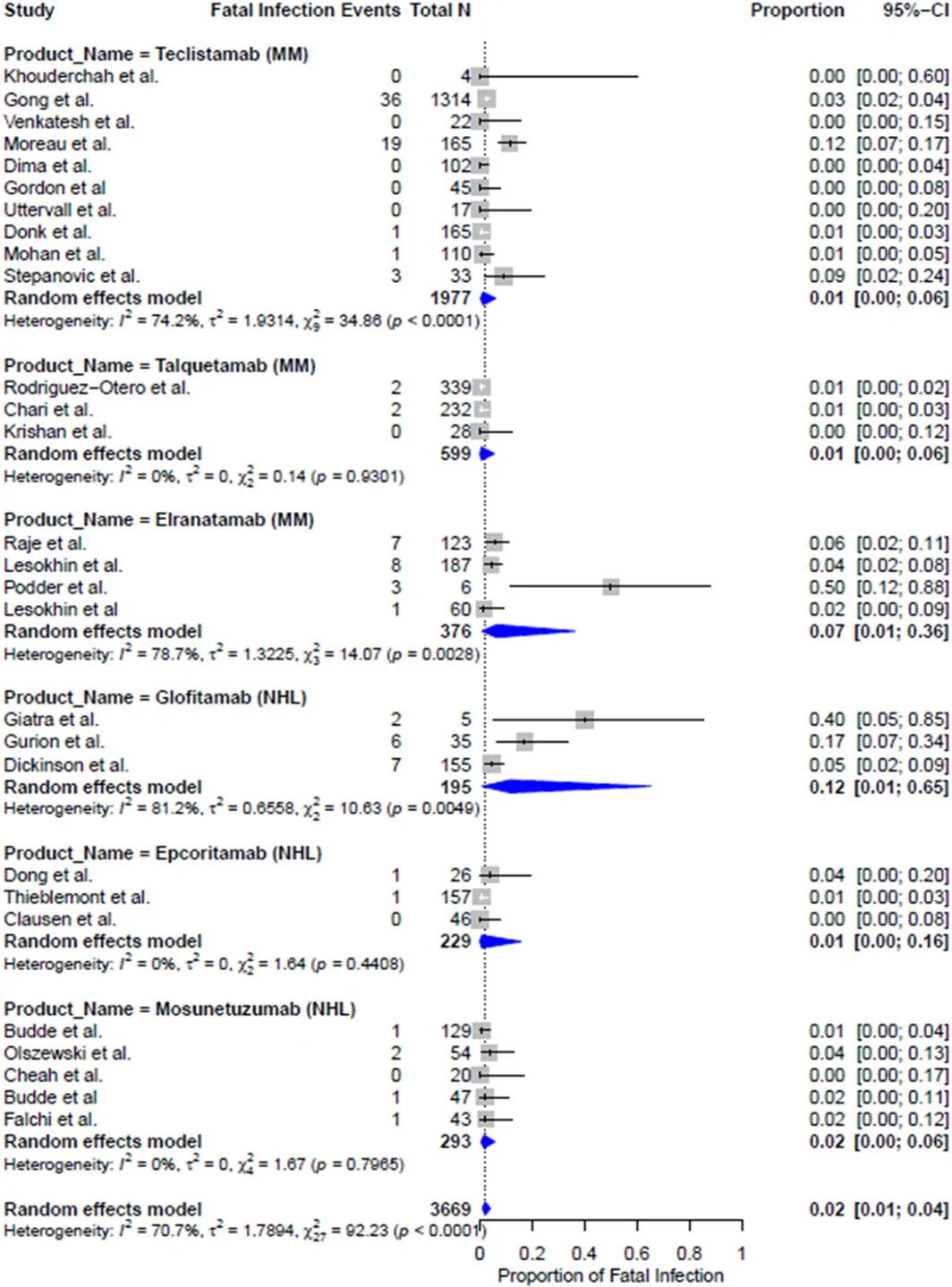
Forest plot depicting fatal infection rates stratified by bispecific antibody product.

##### 3.3.1.1 FAERS Pharmacovigilance

Analysis of severe AEs showed that reported infection‐attributable mortality was highest with glofitamab (39.2%; 87/222), followed by teclistamab (30.3%; 69/228), elranatamab (29.5%; 54/183), and epcoritamab (26.2%; 175/668) (Table 5).

**TABLE 5:** Outcomes stratified by BsAb products according to FAERS database.

	Disease	Total AEs	CRS (%)	CRS mortality (%)	ICANS (%)	ICANS mortality (%)	Infections (%)	Infection mortality (%)
Teclistamab	MM	595	166 (27.9)	30 (18.1)	75 (12.6)	27 (36.0)	228 (38.3)	69 (30.3)
Elranatamab	MM	374	93 (24.8)	11 (11.8)	26 (6.9)	7 (26.9)	183 (48.9)	54 (29.5)
Talquetamab	MM	219	81 (36.9)	6 (7.4)	27 (12.3)	4 (14.8)	93 (42.4)	8 (8.6)
Glofitamab	NHL	741	307 (41.4)	80 (26.1)	32 (4.3)	13 (40.6)	222 (29.9)	87 (39.2)
Epcoritamab	NHL	1671	615 (36.8)	114 (18.5)	75 (4.4)	24 (32.0)	668 (39.9)	175 (26.2)
Mosunetuzumab	NHL	271	103 (38.0)	6 (5.8)	2 (0.7)	1 (50.0)	131 (48.3)	28 (21.4)

Abbreviations: AE, Adverse Event; CRS, Cytokine Release Syndrome; ICANS, immune effector cell‐associated neurotoxicity syndrome.

### 3.4 Grade ≥ 3 Infections

#### 3.4.1 Systematic Review and Meta‐Analysis

The pooled incidence of grade ≥ 3 infections was 12% (95% CI, 7%–21%) when all studies were included (Figure 11). When restricted to individual products, it was reported at 10% (95% CI, 5%–18%) (Figure 12). Subgroup analysis by disease revealed a higher incidence in MM (14%–19%) compared with NHL (6%). At the product level, severe infections were most associated with elranatamab (26%; 95% CI, 19%–34%) and teclistamab (14%), whereas talquetamab and mosunetuzumab were associated with very low rates (2%) (Figure 13).

**FIGURE 11:**
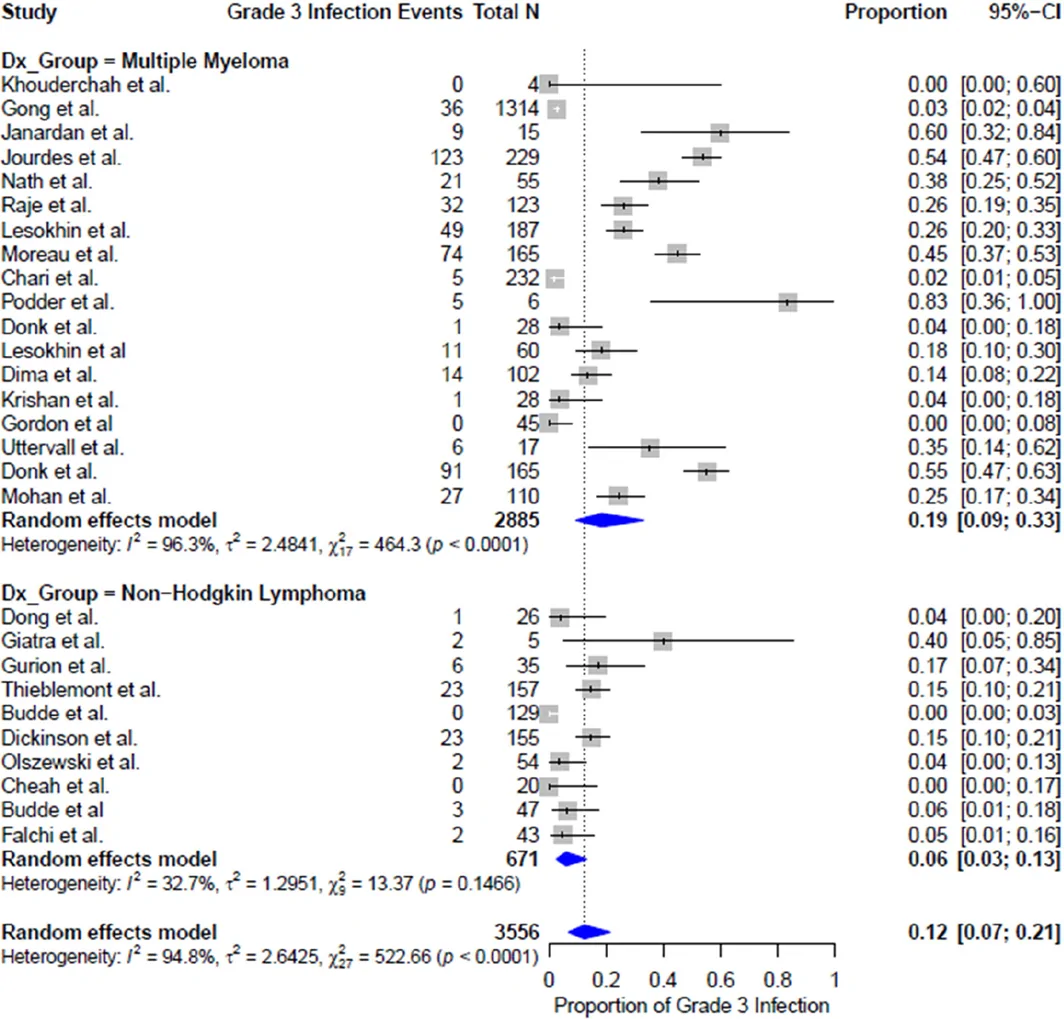
Forest plot showing pooled estimates of grade 3 infection rates across all included studies and cohorts.

**FIGURE 12:**
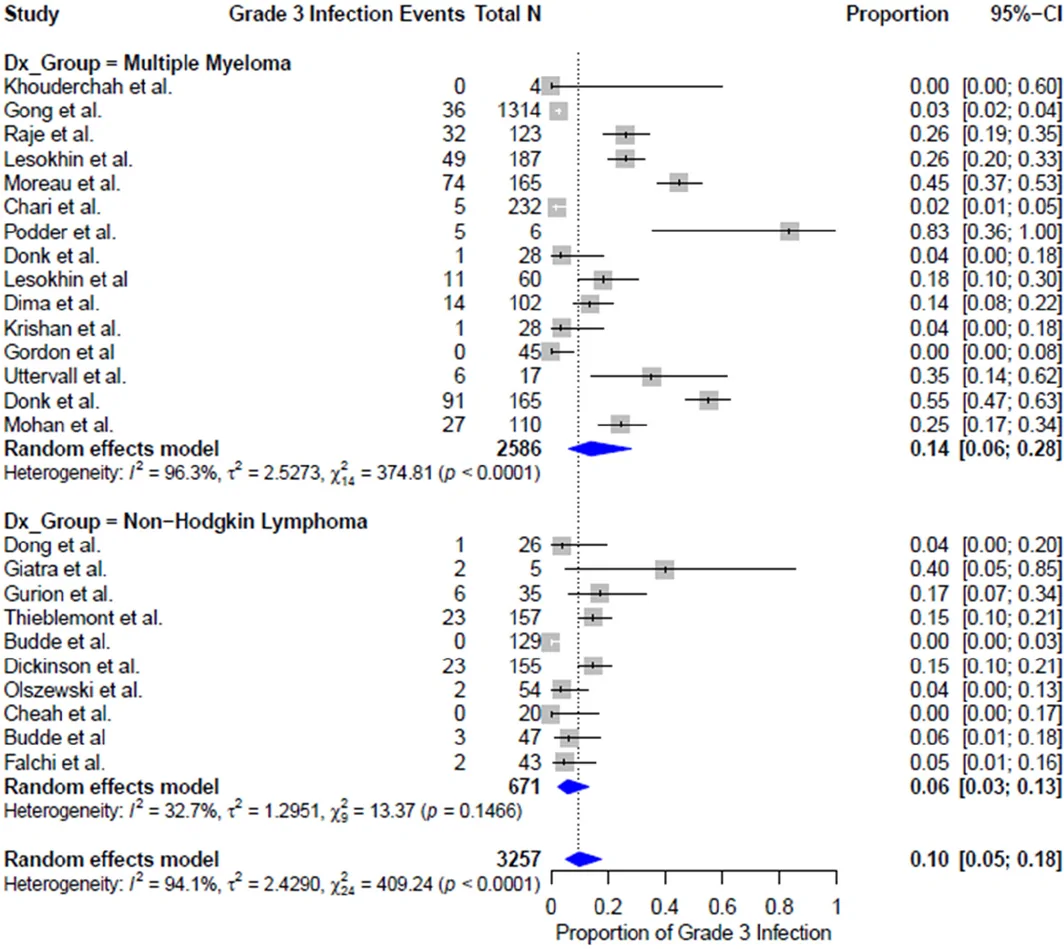
Forest plot of grade 3 infection rates across included studies excluding combination cohort studies, stratified by disease group (Multiple Myeloma and Non‐Hodgkin Lymphoma).

**FIGURE 13:**
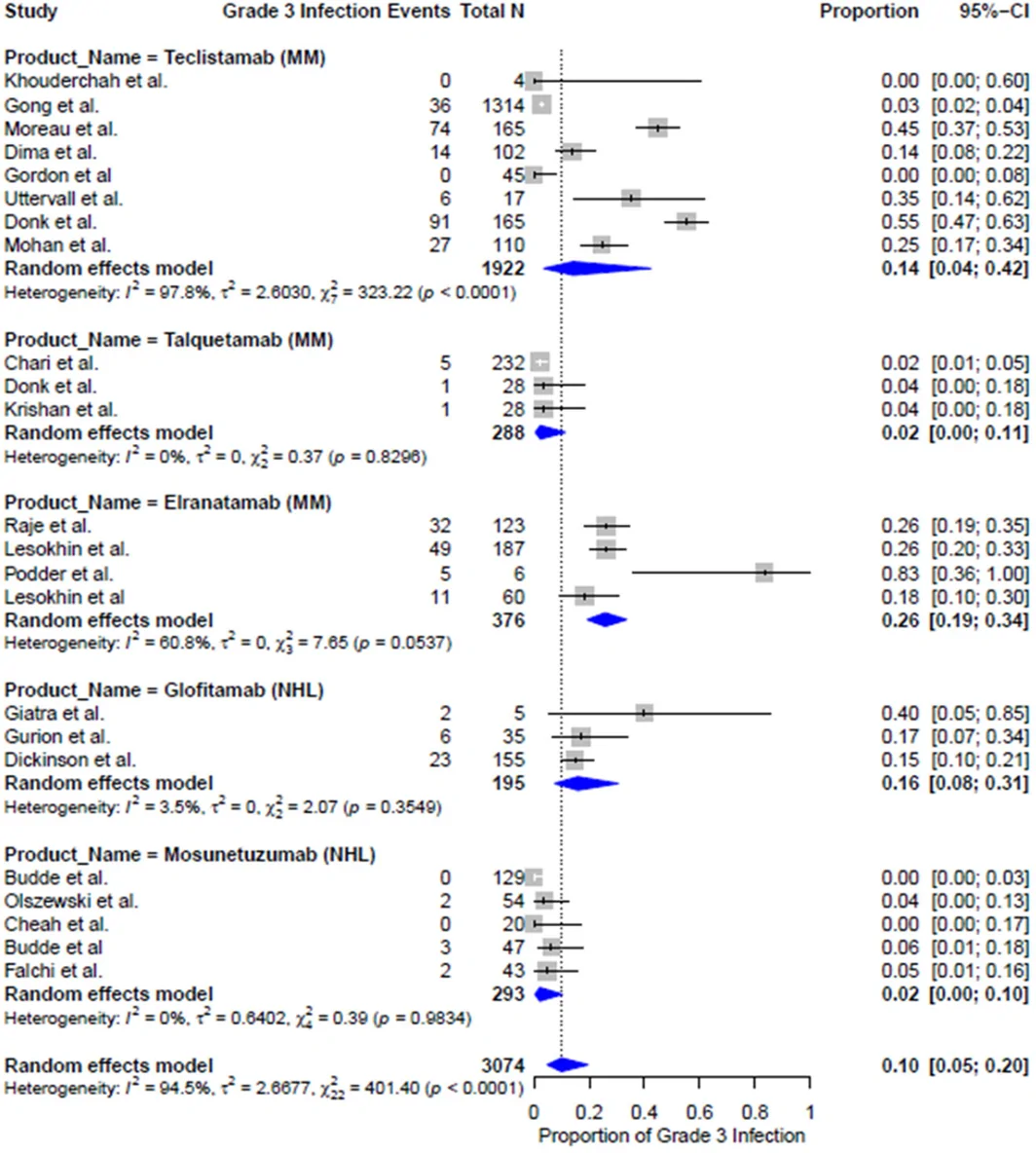
Forest plot depicting CRS infection rates stratified by bispecific antibody product.

### 3.5 Cytokine Release Syndrome (CRS)

#### 3.5.1 Systematic Review and Meta‐Analysis

Any‐grade CRS was frequent, with an overall incidence of 52% (95% CI, 46%–58%) (Figures 14 and 15). MM patients showed higher CRS rates (57%) than NHL patients (47%). At the product level (Figure 16), talquetamab had the highest CRS incidence (67%), followed by teclistamab (58%) and epcoritamab (51%), and mosunetuzumab (40%). Grade ≥ 3 CRS was rare, at 1% overall (Figures 17 and 18). By agent (Figure 19), severe CRS was < 1% for elranatamab, teclistamab (1%), and mosunetuzumab (1%), and higher with talquetamab (5%).

**FIGURE 14:**
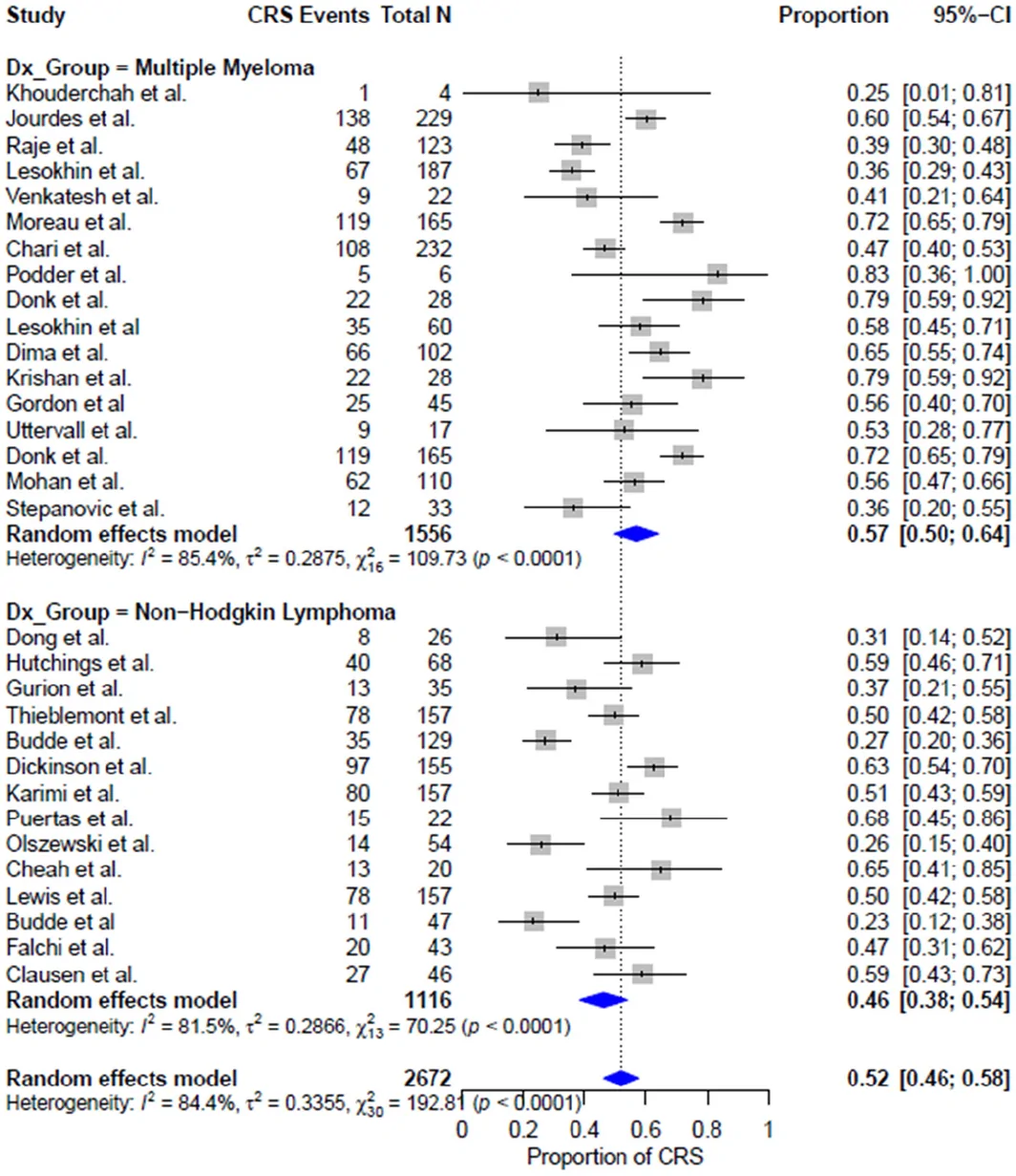
Forest plot showing pooled estimates of grade 3 infection rates across all included studies and cohorts.

**FIGURE 15:**
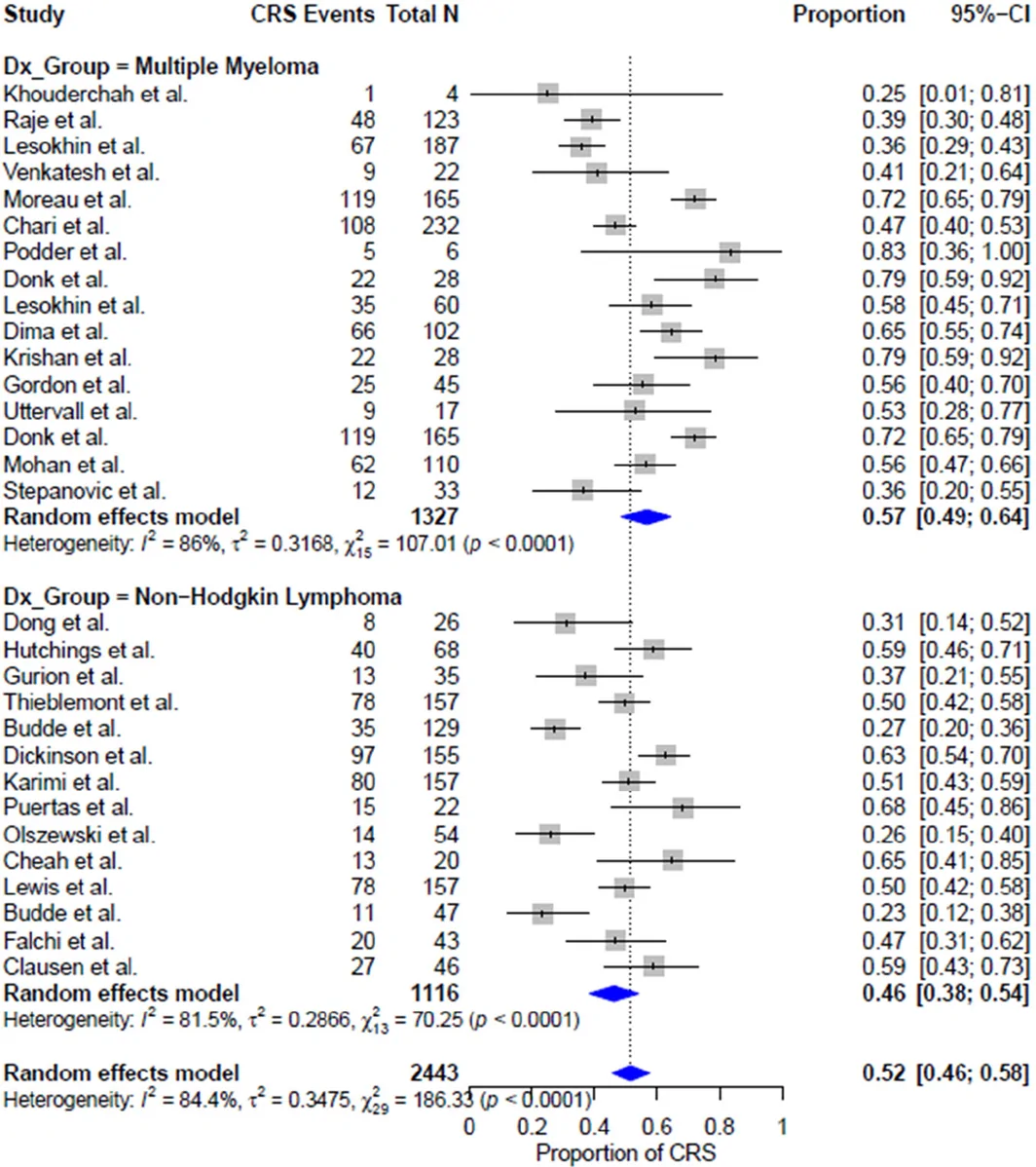
Forest plot of CRS rates across included studies excluding combination cohort studies, stratified by disease group (Multiple Myeloma and Non‐Hodgkin Lymphoma).

**FIGURE 16:**
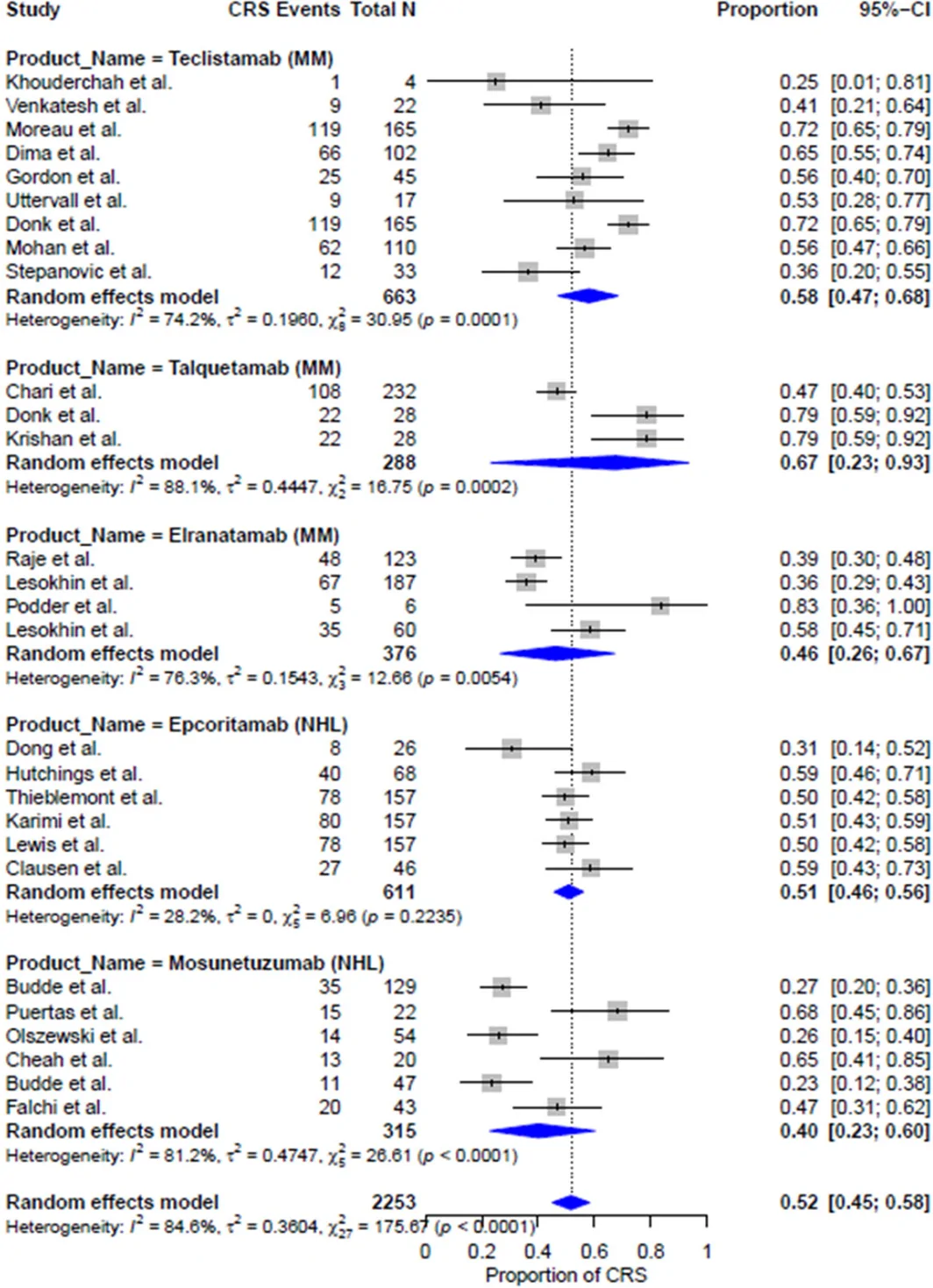
Forest plot depicting CRS rates stratified by bispecific antibody product.

**FIGURE 17:**
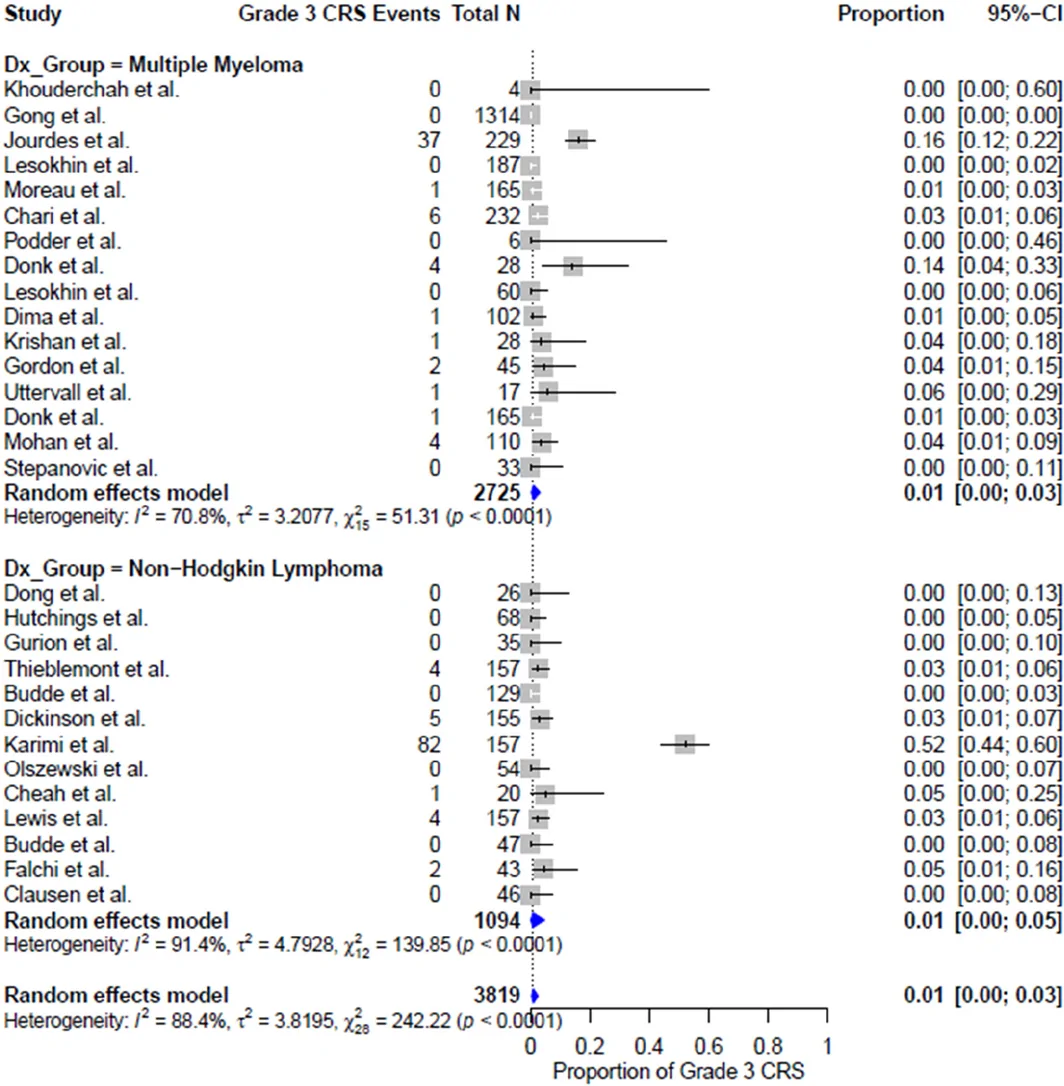
Forest plot showing pooled estimates of grade ≥ 3 CRS rates across all included studies and cohorts.

**FIGURE 18:**
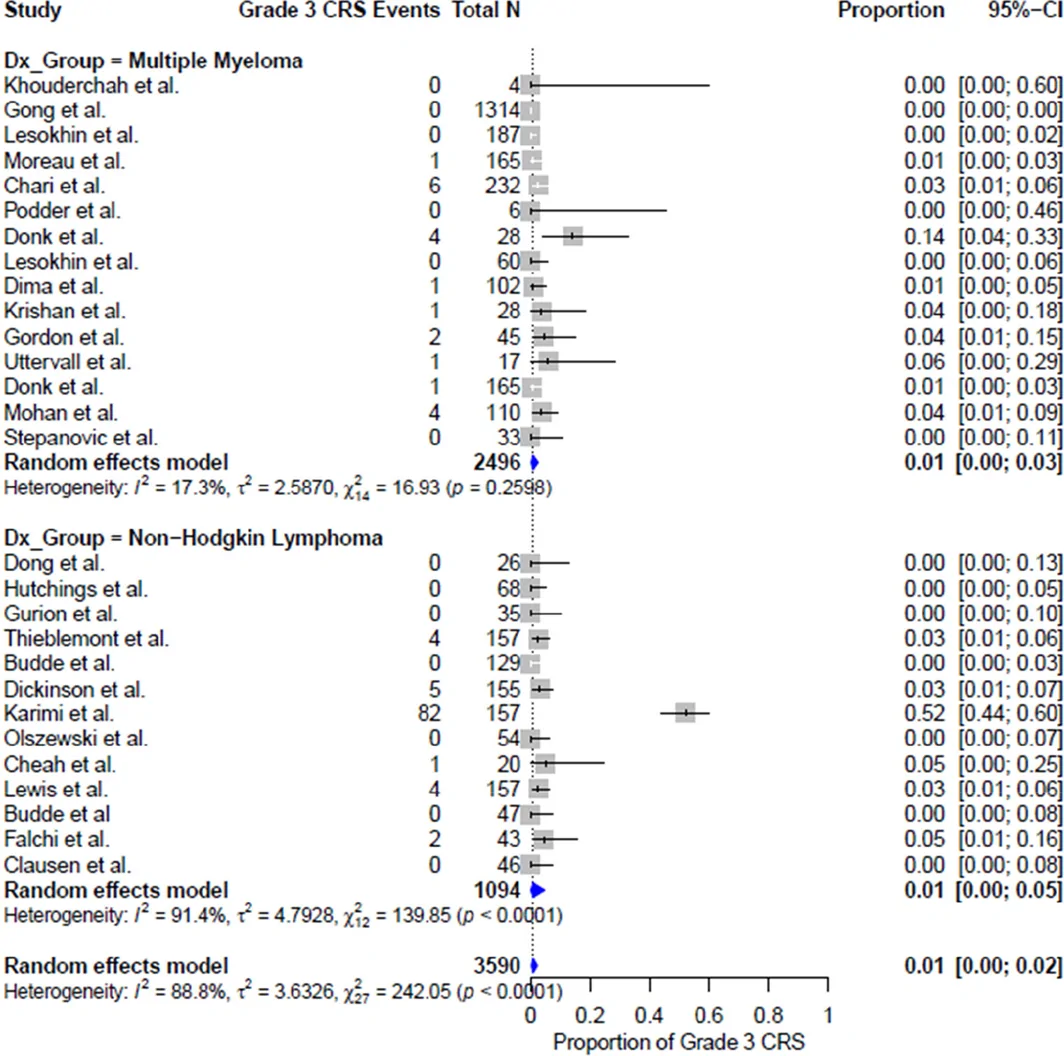
Forest plot of grade 3 CRS rates across included studies excluding combination cohort studies, stratified by disease group (Multiple Myeloma and Non‐Hodgkin Lymphoma).

**FIGURE 19:**
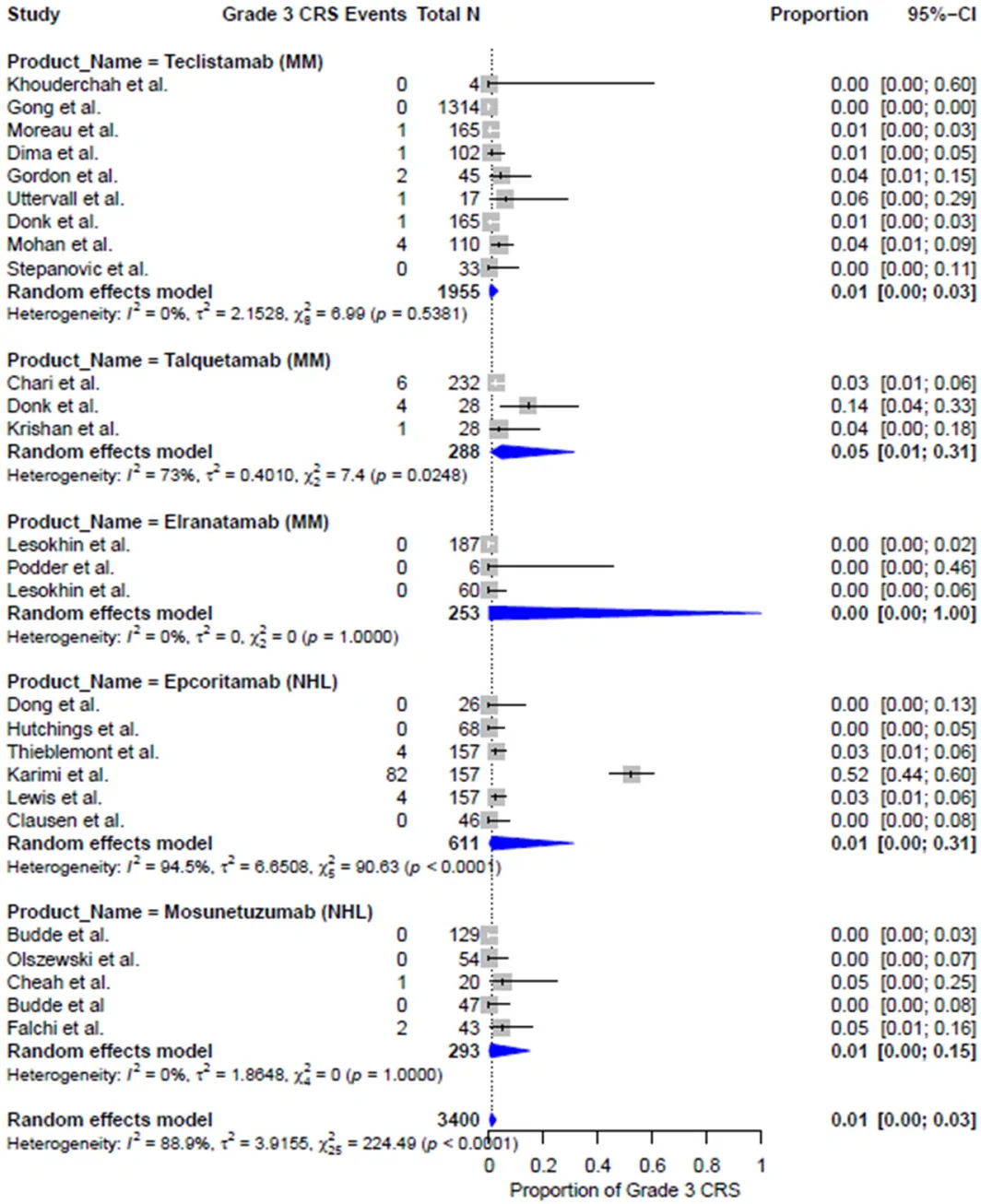
Forest plot depicting grade 3 CRS rates stratified by bispecific antibody product.

#### 3.5.2 FAERS Pharmacovigilance

Glofitamab (ROR = 1.63 [95% CI, 1.40–1.91]) and epcoritamab (ROR = 1.48 [95% CI, 1.30–1.68]) were associated with higher reporting odds for CRS than other BsAbs, while elranatamab (ROR = 0.62 [95% CI, 0.49–0.79]), talquetamab (ROR = 0.43 [95% CI, 0.34–0.55]), and teclistamab (ROR = 0.69 [95% CI, 0.58–0.83]) showed significantly lower CRS reporting. Similarly, reported CRS‐related mortality was highest with glofitamab (26.1%; 80/307), followed by epcoritamab (18.5%; 114/615) and teclistamab (18.1%; 30/166) (Figure 23, Table 5).

#### 3.5.3 ICANS (Neurotoxicity)

##### 3.5.3.1 Systematic Review and Meta‐Analysis

The pooled ICANS Incidence was 5% (95% CI, 3%–8%) (Figure 20 and Figure 21), with a similar rate across NHL (5%) and MM (6%). Agent‐specific ICANS rates ranged from 1% (mosunetuzumab) to 7% (teclistamab) (Figure 22); rates were moderate with epcoritamab (6%) and talquetamab (4%) and lower with elranatamab (3%)

**FIGURE 20:**
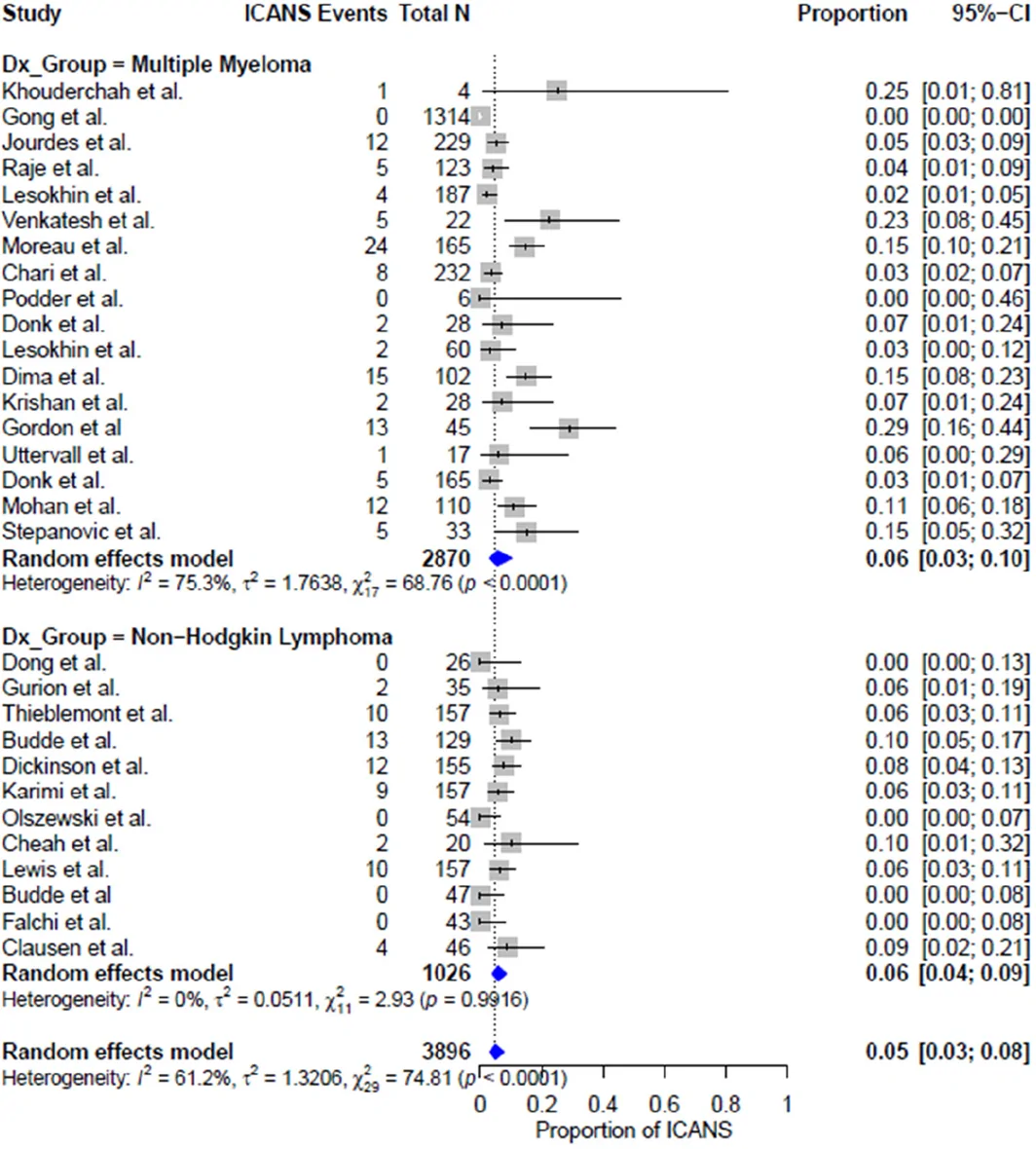
Forest plot showing pooled estimates of ICAN rates across all included studies and cohorts.

**FIGURE 21:**
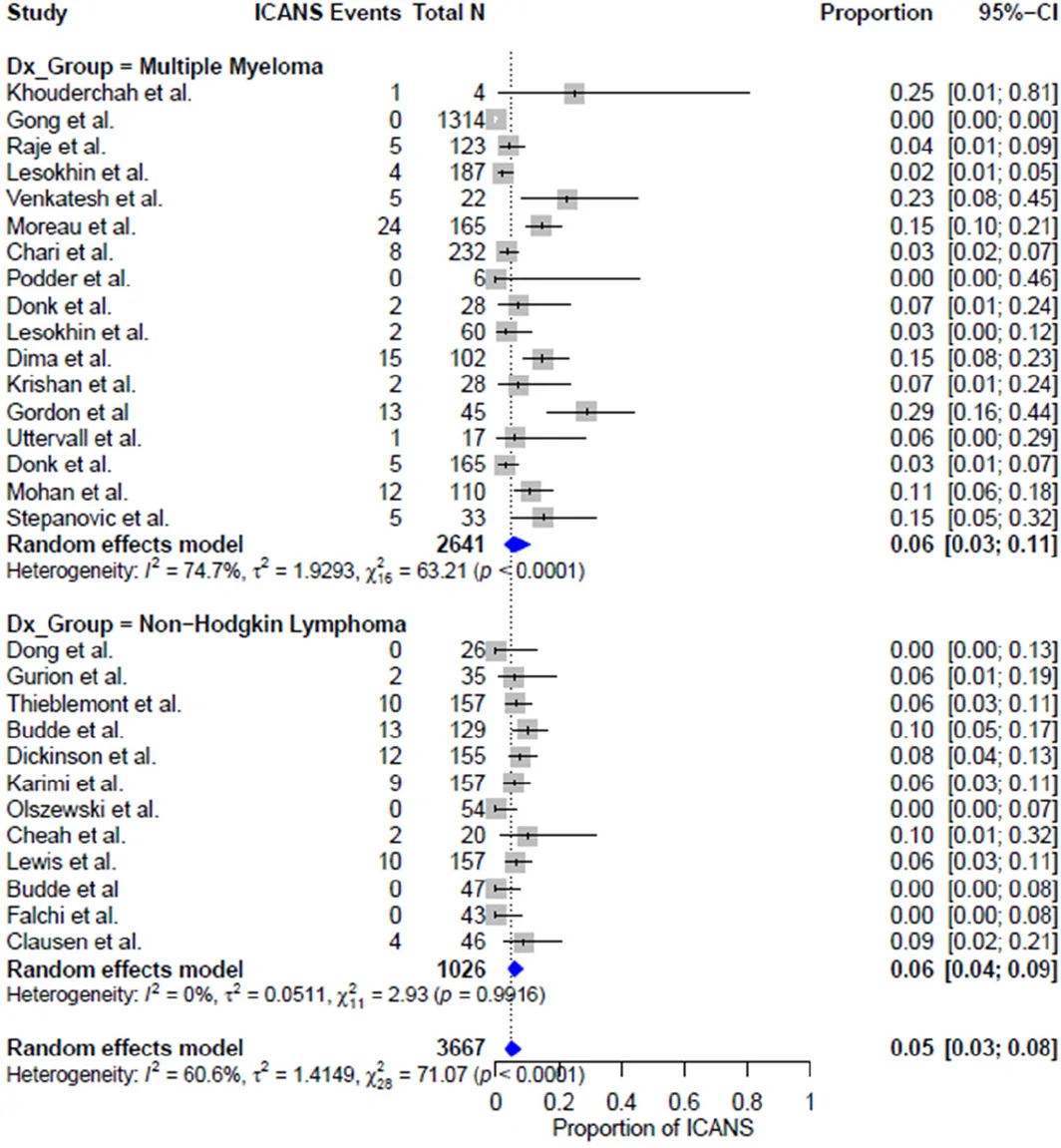
Forest plot of ICAN rates across included studies excluding combination cohort studies, stratified by disease group (Multiple Myeloma and Non‐Hodgkin Lymphoma).

**FIGURE 22:**
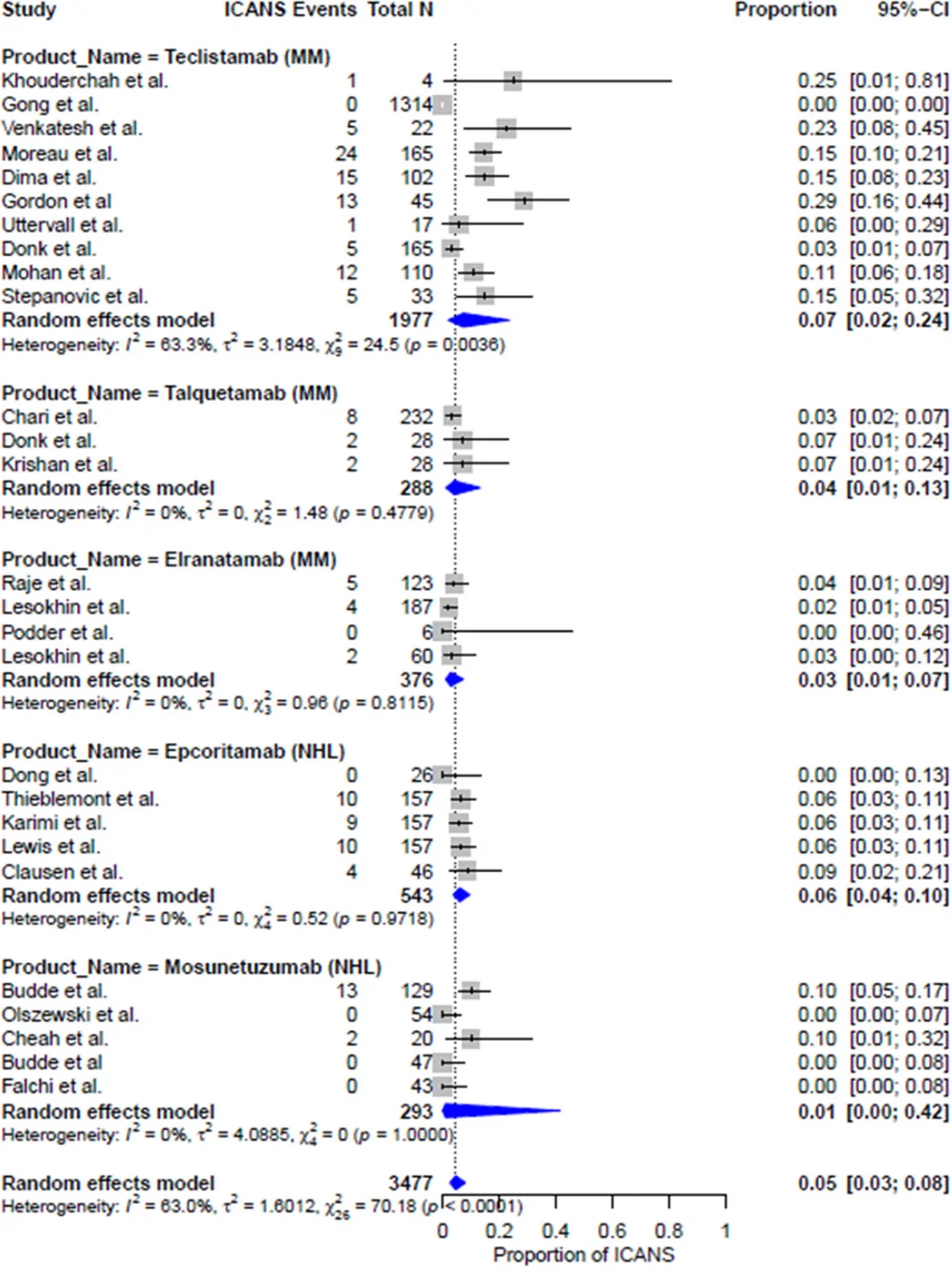
Forest plot depicting ICAN rates stratified by bispecific antibody product.

**FIGURE 23:**
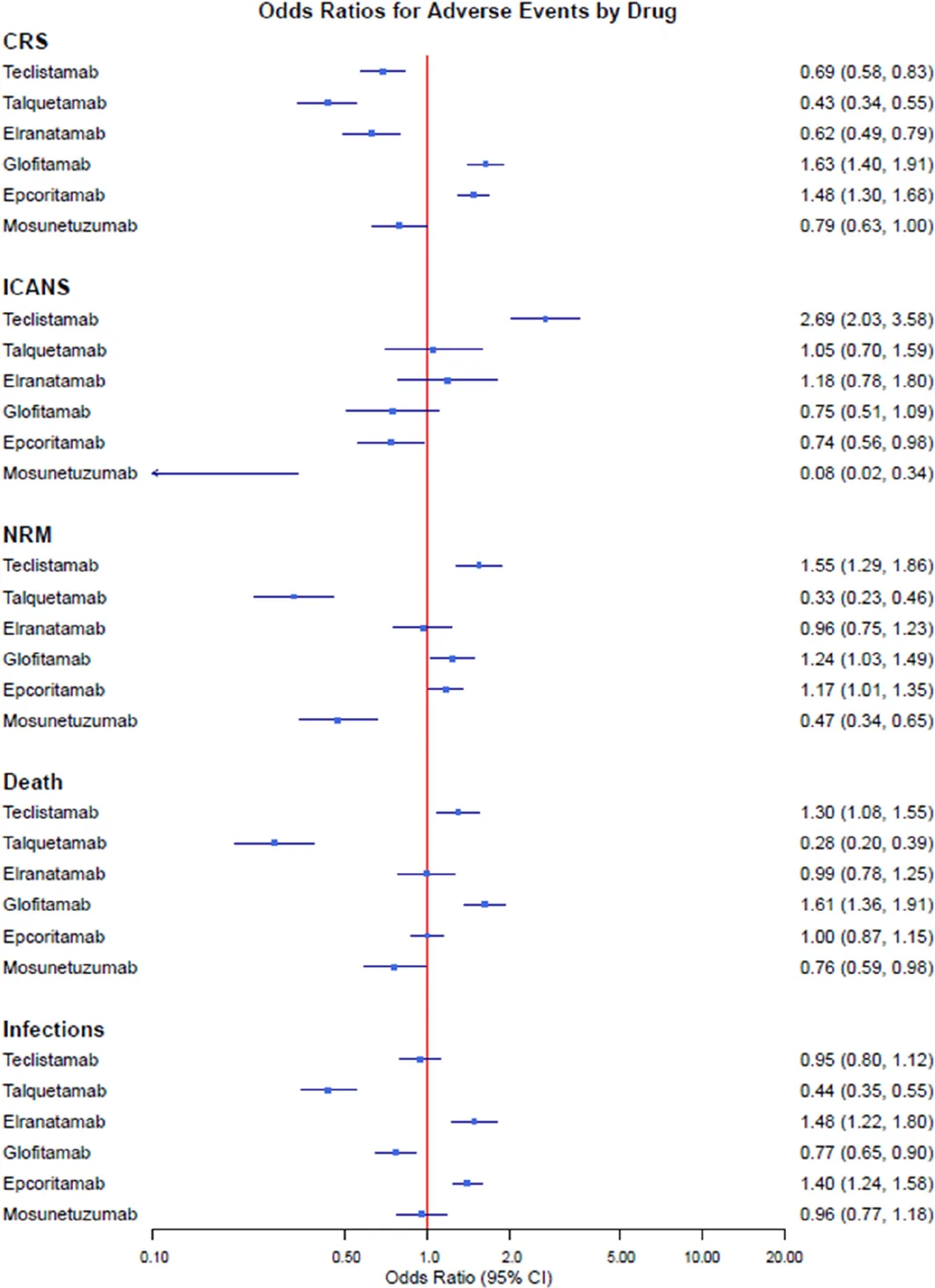
Forest plot showing pooled estimates of fatal infection rates across all included studies and cohorts.

##### 3.5.3.2 FAERS Pharmacovigilance

Teclistamab (ROR = 2.69 [95% CI, 2.03–3.58]) was associated with increased ICANS, while mosunetuzumab and epcoritamab were associated with reduced reporting odds. (ROR = 0.08 [95% CI, 0.02–0.34] and ROR = 0.74 [95% CI, 0.56–0.98], respectively). While the AE reports for ICANS in the FAERS database were notably low, reported ICANS‐attributable mortality was highest with mosunetuzumab (50%; 1/2), followed by glofitamab (40.6%; 13/32) and teclistamab (36%; 27/75) (Figure 23, Table 5).

## 4 Discussion

Our dual methodology incorporates post‐marketing FAERS pharmacovigilance and SRMA to identify bispecific antibodies with convergent and divergent safety signals across clinical trial data and real‐world adverse event reporting, with particular emphasis on fatal outcomes such as NRM, ICANS, and infection‐related mortality. This approach integrates both real‐world outcomes and clinical trial data, thereby quantifying estimates of fatal outcomes associated with these therapies. FAERS analyses in this study are interpreted as identifying toxicity‐associated mortality reporting signals rather than estimating adjudicated non‐relapse mortality.

Among the therapies analyzed, teclistamab demonstrated a high toxicity burden across both analyses. Teclistamab exhibited the highest ICANS incidences, significant rates of infection (particularly grade > 3), and a non‐negligible NRM estimate in the SRMA. These findings were mirrored in FAERS, where teclistamab was associated with the highest reporting odds for NRM and ICANS. This concordance is biologically plausible, as demonstrated in prior studies attributing these findings to profound hypogammaglobulinemia, sustained B‐cell aplasia, T‐cell exhaustion, and impaired antiviral immunity during continuous BCMA‐directed bispecific therapy [8, 48, 49, 50, 51, 52, 53].

Glofitamab stood out across both analytic approaches, especially for severe and fatal toxicity. In the SRMA, analyzing the glofitamab toxicity revealed NRM and fatal infections pooled outcomes at 12% [95% CI, 1%–62%] and 12% [95% CI, 1%–65%], respectively. While these were the highest among the bispecifics, the wide confidence intervals (due to limited study numbers) warrant careful interpretation by clinicians. However, the FAERS analysis reinforced this finding. Glofitamab was associated with increased reporting odds for NRM and overall mortality; furthermore, among fatal adverse event reports, infections and CRS contributed to a substantial proportion of deaths, supporting a convergent signal for serious toxicity outside trial‐selected populations. This highlights the novelty of our approach, which enables the comparison and strengthening of findings using real‐world outcomes, particularly in cases with a limited number of studies. These observations are also consistent with previously published clinical trials and cohort studies reporting CRS and infectious complication burdens [39, 54].

Mosunetuzumab consistently demonstrated lower pooled estimates and lower reporting odds, as seen in both analytic approaches. Low pooled incidences of infections, and ICANS were calculated in the SRMA, alongside an NRM estimate of 4%. FAERS analyses also suggested reduced reporting odds for NRM and neurotoxicity. The reduced incidences and likelihood can be attributed to both treatment‐related and biological factors, such as fixed‐duration therapy and less heavily pretreated lymphoma populations [38, 55].

Interestingly, some discordant signals were also observed between the two analytic methods. This highlights the non‐interchangeable nature of systematic trial synthesis and pharmacovigilance, despite their complementarity for various outcomes [56]. In the SRMA, elranatamab exhibited the highest pooled infection incidence and a notably elevated NRM point estimate, yet this did not translate into increased NRM reporting odds in FAERS. This discrepancy can be explained by shorter post‐approval exposure time and underreporting rather than a true attenuation of risk [57, 58]. Talquetamab similarly demonstrated partial discordance, with high CRS incidence in the SRMA but reduced CRS reporting odds in FAERS. This reflects how patient selection and evolving mitigation strategies to optimize patient outcomes can present as variations between clinical trials and real‐world practice [59].

For epcoritamab, an interesting pattern was documented in both analyses. While concordance was observed for high CRS burden, and high reporting odds in FAERS incidence in the SRMA could not be calculated due to limited studies. The SRMA though suggested lowest non‐relapse mortality despite these immune‐mediated toxicities. These findings highlight how discordance between trial‐based and pharmacovigilance data most often reflects differences in exposure time, population risk, and reporting dynamics rather than contradictory biological effects [60, 61].

Taken together, these findings identify teclistamab and glofitamab as the bispecific antibodies with the most consistent and concerning safety signals across both systematic review and pharmacovigilance analyses, particularly with respect to infection‐related mortality, ICANS, and NRM. With new trials reporting better survival outcomes, bispecific antibodies are increasingly being viewed as an earlier line of therapy [62]. This shift, coupled with prolonged treatment durations, underscores the need for more stringent infection monitoring, including appropriate antimicrobial prophylaxis and standardized immunoglobulin replacement [63]. Incorporation of non‐relapse mortality as a predefined endpoint in future trials and registries will be critical to accurately capture the cumulative toxicity burden of these agents and guide safer therapeutic sequencing [5].

The study has several important limitations that need to be considered when interpreting the results. The pooled incidence proportions were calculated using studies with heterogeneous designs, different patient populations, and follow‐up durations, which limits direct comparison among the agents. Non‐relapse mortality is a time‐dependent outcome, and variation in follow‐up length may bias pooled estimates despite the use of random‐effects models. Because most primary studies did not report time‐to‐event NRM data, a formal time‐to‐event meta‐analysis was not feasible, and pooled NRM should be interpreted as descriptive cumulative burden. Follow‐up duration was unreported in 11 of 38 studies, concentrated in conference abstracts and early‐access cohorts; for these, time‐adjusted estimation was not possible, though their exclusion did not materially change the pooled estimate. This limitation is also particularly relevant when comparing agents with fixed‐duration regimens to those administered continuously, as differential exposure time may contribute to apparent differences in cumulative mortality. Variations in definitions for the outcomes studied can lead to misclassification, which can impact the incidences, thereby affecting the pooled estimates [64]. Analyses using the FAERS database are inherently subject to underreporting bias. Crucially, FAERS does not explicitly capture non‐relapse mortality. NRM was a derived endpoint calculated by excluding reports of disease progression, which likely underestimates the true incidence of toxicity‐related fatalities compared to adjudicated clinical trial data [65]. Additionally, the lack of denominator data means reporting odds ratios reflect disproportionality rather than true incidence or causality [66]. FAERS does not provide information on disease status, prior therapies, or infection prophylaxis. Finally, discordance between trial‐based and FAERS findings may reflect differences in patient selection, duration of exposure, and real‐world practice patterns rather than true biological differences between agents. Accordingly, the results of both approaches should be interpreted as complementary signals rather than definitive risk estimates.

## 5 Conclusion

By combining meta‐analysis with FAERS pharmacovigilance, we identified teclistamab and glofitamab as having the highest burden of non‐relapse mortality and severe infections among currently approved bispecific antibodies. Mosunetuzumab consistently showed lower pooled estimates and lower reporting odds. While trial‐level data and real‐world reporting largely converged for severe toxicities, divergences in other agents underscore the importance of post‐marketing surveillance. Given the risks associated with prolonged treatment, future protocols must prioritize infection management and adopt NRM as a standardized endpoint to guide therapeutic sequencing.

## Author Contributions

All authors contributed to the manuscript and fulfilled the criteria per the uniform requirements set forth by the International Committee of Medical Journal Editors (ICJME) guidelines. All authors have reviewed and approved the final version of the manuscript.

## Funding

The authors have nothing to report.

## Ethics Statement

The authors have nothing to report.

## Consent

The authors have nothing to report.

## Conflicts of Interest

The authors declare no conflicts of interest.

## Supporting information


Supporting Information S1

## Data Availability

The data that support the findings of this study are available in FDA Adverse Event Reporting System (FAERS) at https://www.fda.gov/drugs/fdas‐adverse‐event‐reporting‐system‐faers/fda‐adverse‐event‐reporting‐system‐faers‐public‐dashboard. These data were derived from the following resources available in the public domain: —FDA Adverse Event Reporting System (FAERS), https://www.fda.gov/drugs/fdas‐adverse‐event‐reporting‐system‐faers/fda‐adverse‐event‐reporting‐system‐faers‐public‐dashboard.
