# Supplementary material for: Non‐Relapse Mortality With Bispecific Antibodies in Hematologic Malignancies: A Dual Systematic Review and Pharmacovigilance Analysis
Source: Hematol Oncol. 2026 Oct 16;44(6):e70268. doi: 10.1002/hon.70268 (PMC13674378; doi:10.1002/hon.70268)
Supplement: Supplementary file 1 [file HON-44-e70268-s001.docx]

**Supplementary Online Content**

Abid MB, Kaur M, Mohlere V, Varisco TJ, Ononogbu O. Non-Relapse Mortality with Bispecific Antibodies in Hematologic Malignancies

**This supplementary material has been provided by the authors to give readers additional information about their work.**

**Table S1.** Demographic data at the patient level…………………………………………………………………………… page 2-3

**Table S2.** Outcomes at the patient level ………………………………………………………………………………………. page 4-6

**Table S3.** Outcomes stratified by the BsAb products ………………………………………………………………………page 7

**Table S4.** Agent-level non-relapse mortality and availability of follow-up duration among included studies ………………………….. page 8

**Figure S1.** Correlation Matrix representing Pearson Correlation Coefficient …………………………………page 8

**Figure S2.** Co-occurrence of adverse events ……………………………………………………………………………….. page 9

**Figure S3.** Outcomes of AEs stratified by BsAb products approved for B-cell lymphoma ………………page 10

**Figure S4.** Outcomes of AEs stratified by BsAb products approved for multiple myeloma …………..…page 11

**Methods.** ………………………………………………………………………………………………………………………….……... page 12-13

**References.** ……………………………………………………………………………………………………………………………….page 14

**Data sharing statement.**…………………………………………………………………………………………………………….page 15

**Study protocol.** ………………………………………………………………………………………………………………………... page 16-19

**Table S1.** Patient demographics

| **Characteristics** | **Elranatamab** | **Epcoritamab** | **Glofitamab** | **Mosunetuzumab** | **Talquetamab** | **Teclistamab** | **Total** |
| --- | --- | --- | --- | --- | --- | --- | --- |
| N (%) | 481 (100) | 1936 (100) | 866 (100) | 448 (100) | 552 (100) | 783 (100) | 5066 (100) |
| **Age group** | | | | | | | |
| Median age (yr) | 67 | 68 | 67 | 68 | 65 | 68 | 68 |
| Q1-Q3 | 60 - 74 | 62 - 75 | 56 - 73 | 59 - 74 | 57 - 71 | 61 - 76 | 60 - 74 |
| <18 yrs | 0 (0.0) | 2 (0.1) | 11 (1.3) | 0 (0.0) | 1 (0.2) | 0 (0.0) | 14 (0.3) |
| 18-44 yrs | 16 (3.3) | 61 (3.2) | 57 (6.6) | 23 (5.1) | 10 (1.8) | 9 (1.1) | 176 (3.5) |
| 45-64 yrs | 133 (27.7) | 451 (23.3) | 228 (26.3) | 109 (24.3) | 71 (12.9) | 106 (13.5) | 1098 (21.7) |
| 65-74 yrs | 131 (27.2) | 621 (32.1) | 244 (28.2) | 123 (27.5) | 70 (12.7) | 120 (15.3) | 1309 (25.8) |
| ±75 yrs | 82 (17.0) | 427 (22.1) | 113 (13.0) | 76 (17.0) | 27 (4.9) | 93 (11.9) | 818 (16.1) |
| Not specified | 119 (24.7) | 374 (19.3) | 213 (24.6) | 117 (26.1) | 373 (67.6) | 455 (58.1) | 1651 (32.6) |
| **Sex** | | | | | | | |
| Female | 199 (41.4) | 721 (37.2) | 288 (33.3) | 184 (41.1) | 131 (23.7) | 256 (32.7) | 1779 (35.1) |
| Male | 218 (45.3) | 982 (50.7) | 463 (53.5) | 192 (42.9) | 179 (32.4) | 239 (30.5) | 2273 (44.9) |
| Not Specified | 64 (13.3) | 233 (12.0) | 115 (13.3) | 72 (16.1) | 242 (43.8) | 288 (36.8) | 1014 (20.0) |
| **Weight group** | | | | | | | |
| <40 kg | 5 (1.0) | 6 (0.3) | 9 (1.0) | 0 (0.0) | 0 (0.0) | 1 (0.1) | 21 (0.4) |
| 40-60 kg | 96 (20.0) | 246 (12.7) | 84 (9.7) | 35 (7.8) | 36 (6.5) | 56 (7.2) | 553 (10.9) |
| 61-80 kg | 102 (21.2) | 573 (29.6) | 152 (17.6) | 124 (27.7) | 62 (11.2) | 82 (10.5) | 1095 (21.6) |
| >80 kg | 60 (12.5) | 344 (17.8) | 105 (12.1) | 90 (20.1) | 48 (8.7) | 66 (8.4) | 713 (14.1) |
| Not Specified | 218 (45.3) | 767 (39.6) | 516 (59.6) | 199 (44.4) | 406 (73.6) | 578 (73.8) | 2684 (53.0) |
| **Case Priority** | | | | | | | |
| 30-Day | 0 (0.0) | 0 (0.0) | 0 (0.0) | 1 (0.2) | 0 (0.0) | 0 (0.0) | 1 (0.0) |
| Direct | 3 (0.6) | 8 (0.4) | 3 (0.3) | 2 (0.4) | 23 (4.2) | 40 (5.1) | 79 (1.6) |
| Expedited | 419 (87.1) | 1873 (96.7) | 759 (87.6) | 430 (96.0) | 300 (54.3) | 485 (61.9) | 4266 (84.2) |
| Non-Expedited | 59 (12.3) | 55 (2.8) | 104 (12.0) | 15 (3.3) | 229 (41.5) | 258 (33.0) | 720 (14.2) |

**Table S2.** Patient-level immune-related toxicities, infections, and NRM stratified by BsAb product

| **Characteristics** | **Elranatamab** | **Epcoritamab** | **Glofitamab** | **Mosunetuzumab** | **Talquetamab** | **Teclistamab** | **Total** |
| --- | --- | --- | --- | --- | --- | --- | --- |
| N (%) | 481 (100) | 1936 (100) | 866 (100) | 448 (100) | 552 (100) | 783 (100) | 5066 (100) |
| **CRS** | | | | | | | |
| Yes | 93 (19.3) | 615 (31.8) | 307 (35.5) | 103 (23.0) | 81 (14.7) | 166 (21.2) | 1365 (26.9) |
| No | 388 (80.7) | 1321 (68.2) | 559 (64.5) | 345 (77.0) | 471 (85.3) | 617 (78.8) | 3701 (73.1) |
| **ICANS** | | | | | | | |
| Yes | 26 (5.4) | 75 (3.9) | 32 (3.7) | 2 (0.4) | 27 (4.9) | 75 (9.6) | 237 (4.7) |
| No | 455 (94.6) | 1861 (96.1) | 834 (96.3) | 446 (99.6) | 525 (95.1) | 708 (90.4) | 4829 (95.3) |
| **Infections** | | | | | | | |
| Yes | 183 (38.0) | 668 (34.5) | 222 (25.6) | 131 (29.2) | 93 (16.8) | 228 (29.1) | 1525 (30.1) |
| No | 298 (62.0) | 1268 (65.5) | 644 (74.4) | 317 (70.8) | 459 (83.2) | 555 (70.9) | 3541 (69.9) |
| **NRM** | | | | | | | |
| Yes | 82 (17.0) | 367 (19.0) | 175 (20.2) | 43 (9.6) | 39 (7.1) | 183 (23.4) | 889 (17.5) |
| No | 15 (3.1) | 26 (1.3) | 61 (7.0) | 31 (6.9) | 1 (0.2) | 5 (0.6) | 139 (2.7) |
| No death | 384 (79.8) | 1543 (79.7) | 630 (72.7) | 374 (83.5) | 512 (92.8) | 595 (76.0) | 4038 (79.7) |
| **HLH** | | | | | | | |
| Yes | 0 (0.0) | 0 (0.0) | 0 (0.0) | 0 (0.0) | 1 (0.2) | 1 (0.1) | 2 (0.0) |
| No | 481 (100.0) | 1936 (100.0) | 866 (100.0) | 448 (100.0) | 551 (99.8) | 782 (99.9) | 5064 (100.0) |
| **Death** | | | | | | | |
| Yes | 97 (20.2) | 393 (20.3) | 236 (27.3) | 74 (16.5) | 40 (7.2) | 188 (24.0) | 1028 (20.3) |
| No | 384 (79.8) | 1543 (79.7) | 630 (72.7) | 374 (83.5) | 512 (92.8) | 595 (76.0) | 4038 (79.7) |
| **Disabled** | | | | | | | |
| Yes | 8 (1.7) | 30 (1.5) | 1 (0.1) | 3 (0.7) | 4 (0.7) | 9 (1.1) | 55 (1.1) |
| No | 473 (98.3) | 1906 (98.5) | 865 (99.9) | 445 (99.3) | 548 (99.3) | 774 (98.9) | 5011 (98.9) |
| **Hospitalized** | | | | | | | |
| Yes | 279 (58.0) | 1391 (71.8) | 359 (41.5) | 234 (52.2) | 138 (25.0) | 201 (25.7) | 2602 (51.4) |
| No | 202 (42.0) | 545 (28.2) | 507 (58.5) | 214 (47.8) | 414 (75.0) | 582 (74.3) | 2464 (48.6) |
| **Life Threatening** | | | | | | | |
| Yes | 45 (9.4) | 174 (9.0) | 29 (3.3) | 14 (3.1) | 16 (2.9) | 31 (4.0) | 309 (6.1) |
| No | 436 (90.6) | 1762 (91.0) | 837 (96.7) | 434 (96.9) | 536 (97.1) | 752 (96.0) | 4757 (93.9) |
| **Non-serious** | | | | | | | |
| Yes | 47 (9.8) | 39 (2.0) | 126 (14.5) | 77 (17.2) | 208 (37.7) | 141 (18.0) | 638 (12.6) |
| No | 434 (90.2) | 1897 (98.0) | 740 (85.5) | 371 (82.8) | 344 (62.3) | 642 (82.0) | 4428 (87.4) |
| **Other outcomes** | | | | | | | |
| Yes | 190 (39.5) | 1231 (63.6) | 392 (45.3) | 157 (35.0) | 209 (37.9) | 394 (50.3) | 2573 (50.8) |
| No | 291 (60.5) | 705 (36.4) | 474 (54.7) | 291 (65.0) | 343 (62.1) | 389 (49.7) | 2493 (49.2) |
| **Required intervention** | | | | | | | |
| Yes | 1 (0.2) | 0 (0.0) | 0 (0.0) | 0 (0.0) | 3 (0.5) | 9 (1.1) | 13 (0.3) |
| No | 480 (99.8) | 1936 (100.0) | 866 (100.0) | 448 (100.0) | 549 (99.5) | 774 (98.9) | 5053 (99.7) |

**Table S3.** Outcomes stratified by BsAb products

|  | Disease | Total AEs | CRS (%) | CRS Mortality (%) | ICANS (%) | ICANS Mortality (%) | Infections (%) | Infection Mortality (%) |
| --- | --- | --- | --- | --- | --- | --- | --- | --- |
| Teclistamab | MM | 595 | 166 (27.9) | 30 (18.1) | 75 (12.6) | 27 (36.0) | 228 (38.3) | 69 (30.3) |
| Elranatamab | MM | 374 | 93 (24.8) | 11 (11.8) | 26 (6.9) | 7 (26.9) | 183 (48.9) | 54 (29.5) |
| Talquetamab | MM | 219 | 81 (36.9) | 6 (7.4) | 27 (12.3) | 4 (14.8) | 93 (42.4) | 8 (8.6) |
| Glofitamab | NHL | 741 | 307 (41.4) | 80 (26.1) | 32 (4.3) | 13 (40.6) | 222 (29.9) | 87 (39.2) |
| Epcoritamab | NHL | 1671 | 615 (36.8) | 114 (18.5) | 75 (4.4) | 24 (32.0) | 668 (39.9) | 175 (26.2) |
| Mosunetuzumab | NHL | 271 | 103 (38.0) | 6 (5.8) | 2 (0.7) | 1 (50.0) | 131 (48.3) | 28 (21.4) |
| *AE -Adverse Event, CRS – Cytokine Release Syndrome, ICANS - immune effector cell-associated neurotoxicity syndrome* | | | | | | | | |

**Table S4: Agent-level non-relapse mortality and availability of follow-up duration among included studies.**

| **Bispecific antibody** | **Treatment schedule** | **Studies, n** | **Pooled NRM, % (95% CI)** | **Studies reporting follow-up, n/N** | **Median follow-up, months (range)** |
| --- | --- | --- | --- | --- | --- |
| Teclistamab | Continuous | 9 | 4 (1–12) | 6/9 | 3.8 (3.1–23.0) |
| Elranatamab | Continuous | 4 | 9 (2–31) | 2/4 | 10.6 (6.4–14.7) |
| Talquetamab | Continuous | 2 | 1 (0–2) | 2/2 | 13.8 (11.7–15.9) |
| Glofitamab | Fixed | 3 | 12 (4–30) | 2/3 | 23.3 (12.6–34.0) |
| Epcoritamab | Continuous | 4 | 1 (1–4) | 3/4 | 10.2 (4.0–10.7) |
| Mosunetuzumab | Fixed | 5 | 4 (2–7) | 4/5 | 8.9 (2.5–23.3) |
| *Abbreviations: NRM, non-relapse mortality; CI, confidence interval; n/N, number of studies reporting follow-up out of the total contributing to the estimate.*  Pooled NRM estimates were derived from random-effects meta-analyses of single proportions (generalized linear mixed model, logit transformation) and correspond to the agent-level estimates in Figure 2C.  Median follow-up (range) is calculated only among studies reporting it; follow-up duration was not reported in *11 of 38* included studies, concentrated in conference abstracts and early-access cohorts.  Treatment schedule reflects approved dosing: *continuous* = administered until disease progression (teclistamab, elranatamab, talquetamab, epcoritamab); *fixed-duration* = glofitamab (12 cycles) and mosunetuzumab (up to 8–17 cycles, response-adapted).  A meta-regression across all follow-up–reporting studies (k=21) showed no significant association between follow-up duration and NRM (odds ratio 1.03 per additional month; 95% CI 0.95–1.12; p=0.49). Pooled NRM did not differ significantly between continuous and fixed-duration schedules (3.1% vs 5.6%; p=0.43), and restricting the overall estimate to follow-up–reporting studies left it materially unchanged (3.6% vs 4.0%). | | | | | |

**Figure S1.** Correlation Matrix representing Pearson Correlation Coefficient

**Figure S2.** Co-occurrence of adverse events

**Figure S3.** Outcomes of AEs stratified by BsAb products approved for B-cell Non-Hodgkin lymphoma

**Figure S4.** Outcomes of AEs stratified by BsAb products approved for relapsed/refractory multiple myeloma

**Methods:**

### Data source:

This study acquired original data from Q3 of 2022 to Q3 of 2024 from the FAERS database downloaded from the FDA public database. The FDA Adverse Event Reporting System (FAERS) is a global surveillance platform, encompassing both voluntary safety reports and mandatory regulatory submissions from healthcare providers, consumers, and manufacturers. Each FAERS report comprises details like administrative data (country, reporter's occupation, year of reporting), patient demographics (age, sex), drug administration specifics (indication, dosage, administration route), adverse event (AE) occurrence date, and outcomes (such as death).

Duplicate reports within FAERS can arise mainly through two routes: through common follow-up reports for the same drug-reaction pair, which share the same case number but have distinct case versions, and secondly, due to the submission of the same event under different case numbers. This second scenario often occurs because of challenges in linking follow-up reports to their original entries and reports by different sources (such as health professionals, manufacturers, and consumers) for the same event. To address suspected duplicates, we implemented a filtering algorithm, targeting reports with identical age, sex, event date, and country of occurrence. Among the identified toxicities, we searched for unique instances, and unequivocal duplicates were removed for accuracy.

We screened the FAERS using the Quarterly extract files for reports on “teclistamab,” “talquetamab,” “elranatamab,” “glofitamab,” “epcoritamab,” and “mosunetuzumab,” covering the period from September 1, 2022, essentially starting from the FDA approval for each of the BsAb product to September 30, 2024. We employed an unbiased filtering approach using medDRA terms and selected medDRA reaction terms that had at least one associated report in BsAb therapy. Moreover, we excluded any suspected instances of recurrent infections to maintain the integrity of our analysis. We identified reactions among unique case reports. For disproportionality analysis, each unique case was counted only once. The FAERS is a publicly accessible and anonymized database, hence institutional review board approval and informed consent were not required.

**Statistical Analysis:**

To identify disproportionality (higher-than-expected frequency of adverse event [AE] reporting), we used the reporting odds ratio (ROR) algorithms. The parameters required for these algorithms were calculated using a 2 × 2 columnar table. The specific formulas for the algorithms and signal detection criteria are available. The AE signals were included in the statistical tables only if they met the algorithm criteria. Statistical analyses were performed using Microsoft Excel 2021 and R version 4.4.0 software. Graphs were created using the “ggplot2” package in R software.

Our analysis involved a disproportionality approach, comparing the reporting incidence of adverse events, a method known as case/non-case analysis. For signal detection, used Reporting Odds Ratio (ROR) as primary analysis. These tools are designed to identify whether a specific drug-AE pairing is reported more frequently than expected, with 'expected' defined as the occurrence of the AE with any other drug in the entire database. ROR, akin to an odds ratio, serves as an easily understandable frequentist measure, especially useful for those outside the statistics field.

ROR calculations were performed using 2×2 contingency tables with the following structure:

- a: number of reports with target drug and AE of interest
- b: number of reports with target drug but without AE of interest
- c: number of reports with other drugs and AE of interest
- d: number of reports with other drugs but without AE of interest

The ROR was calculated as: ROR = (a × d) / (b × c). The 95% confidence intervals for ROR were calculated using logarithmic transformation with the standard error formula: SE(log ROR) = √(1/a + 1/b + 1/c + 1/d). For NRM, cases with no reported death were treated as having no non-relapse mortality (NRM).

Continuous variables were expressed as the median with interquartile range (IQR). Forest plots were generated to visualize odds ratios and their 95% confidence intervals across all drug-adverse event groups. Correlation analysis was performed to examine relationships between different adverse events based on their reporting patterns across drugs using Pearson correlation coefficients. Visualization included correlation matrices and circular plot to illustrate co-occurrence patterns of adverse events.

**References:**

1. Bate A, Lindquist M, Edwards IR, et al. A Bayesian neural network method for adverse drug reaction signal generation. Eur J Clin Pharmacol. 1998;54(4):315-321. doi:10.1007/s002280050466

2. Bate A, Evans SJW. Quantitative signal detection using spontaneous ADR reporting. Pharmacoepidemiol Drug Saf. 2009;18(6):427-436. doi:10.1002/pds.1742

3. Norén GN, Hopstadius J, Bate A. Shrinkage observed-to-expected ratios for robust and transparent large-scale pattern discovery. Stat Methods Med Res. 2013;22(1):57-69. doi:10.1177/0962280211403604

4. Harpaz R, Dumouchel W, Lependu P, Bauer-Mehren A, Ryan P, Shah NH. Performance of pharmacovigilance signal-detection algorithms for the FDA adverse event reporting system. Clin Pharmacol Ther. 2013;93(6):539-546. doi:10.1038/clpt.2013.24

5. Harpaz R, Dumouchel W, Shah NH, Madigan D, Ryan P, Friedman C. Novel datamining methodologies for adverse drug event discovery and analysis. Clin Pharmacol Ther. 2012;91(6):1010-1021. doi:10.1038/clpt.2012.50

**Data Sharing Statement**

**Data**

Data available: Yes

Data types: Other (please specify)

Additional Information: Data are publicly available via the FDA Adverse Events System

How to access data: <https://open.fda.gov/data/faers/>

When available: With publication

**Supporting Documents**

Document types: Other (please specify)

Additional Information: Selection criteria applied to the FAERS system will be shared

How to access documents: In the method section

When available: With publication

**Additional Information**

Who can access the data: public

Types of analyses: all analysis

Mechanisms of data availability: it is publicly available

**Study Protocol**

**Title:** Immune-Mediated Toxicities and Non-Relapse Mortality with Bispecific Antibodies for Lymphoma and Multiple Myeloma: A Pharmacovigilance Analysis of the FDA Reporting System

**PI:** Onye Ononogbu PharmD, MPH, BCOP

**PI:** Muhammad Bilal Abid, MD, MS, FACP, MRCP, FRCP

**Project Overview:** Bispecific antibodies (BsAb) have shown promising response rates across hematologic malignancies. However, BsAbs are also associated with an increased risk of infections. To better understand these risks, we will analyze adverse events data using the FAERS FDA database.

**Aim**: To determine the risk profile of adverse events associated with bispecific antibodies in the treatment of Multiple Myeloma and Lymphoma, as reported in the FAERS FDA database.

**Scientific Impact:**

The findings from the planned analysis will underscore the importance of pharmacovigilance strategies for patients undergoing BsAb therapy and will have significant clinical implications in terms of personalizing care for patients. The results will potentially allow clinicians and centers to consider the unique risk profile of each BsAb product when selecting treatment options, implement stringent monitoring protocols, and educate patients about potential risks. Healthcare facilities would be able to ensure adequate resources and staff training for managing adverse events, especially when administering higher-risk therapies, leading to safer, and effective BsAb therapies.

**Hypothesis:**

Utilizing the FAERS FDA database, this study hypothesizes that while these therapies demonstrate efficacy, they also pose a heightened risk of significant adverse events, particularly infections, leading to increased mortality rates. The investigation will focus on categorizing these events by severity (all grades and grades ≥3) to assess the risk profile and inform clinical decision-making. This analysis will provide time-sensitive and product-specific AE data, unearth crucial differences in the risk profiles of CRS, ICANS, and IEC-HS across the 6 BsAb therapies, and allow centers to personalize care.

**Research Question:**

- What is the non-relapse mortality (NRM) rate and the causes of death in patients with relapsed / refractory (R/R) lymphoma and multiple myeloma treated with bispecific antibodies (BsAb), and how do these outcomes vary across different disease subtypes, bispecific antibody products, and target antigen?
- How do immune-mediated toxicities (CRS, ICANS, and ICE-HS) and infections with BsAbs differentially impact mortality and NRM?

**PICOS Formulation:**

**Population (P):**

- **Population:**
  Adult patients diagnosed with the following hematological malignancies:
  - Follicular lymphoma (FL)
  - Diffuse Large B-cell lymphoma (DLBCL)
  - Multiple myeloma (MM)
- **Intervention:**
  Treatment with bispecific antibodies, including agents approved by the U.S. Food and Drug Administration (FDA) for lymphoma or multiple myeloma.
  - Teclistamab
  - Elranatamab
  - Talquetamab
  - Glofitamab
  - Epcoritamab
  - Mosunetuzumab
- **Comparison:**
  Subgroup analyses based on:
  - Disease subtype (FL, DLBCL, MM)
  - Specific bispecific antibody products.
  - Treatment setting (clinical trials vs. real-world practice)
- **Outcomes:**

**Primary Outcome:** Non-relapse mortality (NRM) rates. Cumulative incidence of NRM. NRM will be defined as death without preceding disease relapse/progression. Relapse and progression are competing events.

**Secondary Outcomes:** Causes of death in NRM cases, overall survival, PFS, and incidence of treatment-related adverse events as listed below.

**Adverse Events (AEs) Analysis:**

- The current analysis will focus on the following adverse events:
  - Cytokine Release Syndrome (CRS): All Grades, Grade ≥ 3
  - Immune Effector Cell-Associated Neurotoxicity Syndrome (ICANS): All Grades, Grade ≥ 3
  - Immune Effector Cell-Associated Hemophagocytic Lymphohistiocytosis (HLH) Syndrome (IEC-HS): All Grades, Grade ≥ 3
  - Infections and Infestations: All Grades, Grade ≥ 3
  - Mortality
- **Study Design:**
  Inclusion of both clinical trials and real-world observational studies to capture a broad perspective on BsAb safety profile.

**Search Strategy:**

1. **Data Extraction:**
   - We will extract data for the following 6 drugs from the FAERS FDA database:
     - **Multiple Myeloma (MM):** Teclistamab, Elranatamab, Talquetamab
     - **Diffuse Large B-cell Lymphoma (DLBCL):** Glofitamab, Epcortimab
     - **Follicular Lymphoma (FL):** Monsunetuzumab
2. **Data Cleaning:**
   - Clean the extracted data to ensure the accuracy and reliability of analysis.

**Eligibility Criteria:**

- **Inclusion Criteria:**
  1. Adult patients (≥18 years) with a confirmed diagnosis of large B-cell lymphoma (DLBCL), follicular lymphoma (FL), or multiple myeloma (MM).
  2. Treatment with bispecific antibodies, approved by the FDA.
  3. Data available on non-relapse mortality (NRM) and/or causes of death, CRS, ICANS, IEC-HS, and infections.
  4. Studies conducted in clinical trials or real-world settings, including both phase I-III randomized controlled trials (RCTs) and observational studies.
  5. Data capture up to December 30, 2024.
- **Exclusion Criteria:**
  1. Studies including pediatric patients or other malignancies outside of FL, DLBCL, and MM.

**Data Analysis:**

Data will be pooled using meta-analysis techniques where applicable, and subgroup analyses will be performed based on the comparisons outlined in the PICOS framework. Descriptive summaries will be provided for studies where analysis is not possible.

**Expected Outcomes:**

This review will provide insights into the safety and NRM associated with bispecific antibodies in the real-world setting. It will also identify factors influencing non-relapse mortality and offer recommendations for clinical practice based on treatment setting and patient characteristics.
